# Umbilical cord mesenchymal stem cell-derived apoptotic extracellular vesicles ameliorate cutaneous wound healing in type 2 diabetic mice via macrophage pyroptosis inhibition

**DOI:** 10.1186/s13287-023-03490-6

**Published:** 2023-09-19

**Authors:** Yiming Wang, Lin Jing, Xiao Lei, Zhen Ma, Bei Li, Yuanyuan Shi, Wuyang Zhang, Yuan Li, Hongzhi Zhou, Kaijin Hu, Yang Xue, Yan Jin

**Affiliations:** 1 State Key Laboratory of Military Stomatology & National Clinical Research Center for Oral Diseases & Shaanxi Clinical Research Center for Oral Diseases, Department of Oral Surgery, School of Stomatology, The Fourth Military Medical University, Xi’an, 710032 China; 2 State Key Laboratory of Military Stomatology & National Clinical Research Center for Oral Diseases & Shaanxi International Joint Research Center for Oral Diseases, Center for Tissue Engineering, School of Stomatology, The Fourth Military Medical University, Xi’an, 710032 Shaanxi China; 3 State Key Laboratory of Military Stomatology & National Clinical Research Center for Oral Diseases & Shaanxi Clinical Research Center for Oral Diseases, Department of Orthodontics, School of Stomatology, The Fourth Military Medical University, Xi’an, 710032 Shaanxi China; 4 The College of Life Sciences and Medicine, Northwest University, Xi’an, 710069 Shaanxi China

**Keywords:** Diabetic cutaneous wound, Apoptotic extracellular vesicles, Pyroptosis, Oxidative stress, Cell death regulation

## Abstract

**Background:**

Delayed healing of diabetic cutaneous wounds is one of the most common complications of type 2 diabetes mellitus (T2DM), which can bring great distress to patients. In diabetic patients, macrophages accumulate around skin wounds and produce NLRP3 (NOD-, LRR-, and pyrin domain-containing protein 3) inflammasomes, which in turn undergo pyroptosis and produce inflammatory factors such as interleukin-1β that affect wound healing. Although our previous study revealed that apoptotic extracellular vesicles (ApoEVs) produced from mesenchymal stem cells (MSCs) improve cutaneous wound healing in normal C57BL/6 mice, whether ApoEVs can also improve diabetic wound healing remains unclear.

**Methods:**

Umbilical cord mesenchymal stem cells (UCMSCs) were cultured in vitro and apoptosis was induced. ApoEVs were extracted and identified and used in a T2DM mouse cutaneous wound model to evaluate the efficacy. The inhibitory effect of ApoEVs on macrophage pyroptosis was verified in vivo and in vitro*,* and the level of oxidative stress in macrophages was assessed to explore the mechanism by which ApoEVs play a role.

**Results:**

UCMSC-derived ApoEVs improved skin defect healing in T2DM mice. Moreover, UCMSC-derived ApoEVs inhibited macrophage pyroptosis in T2DM mice in vivo as well as in vitro under high-glucose culture conditions. In addition, we demonstrated that ApoEVs reduce oxidative stress levels, which is a possible mechanism by which they inhibit macrophage pyroptosis.

**Conclusions:**

Our study confirmed that local application of UCMSC-derived ApoEVs improved cutaneous wound healing in T2DM mice. ApoEVs, as products of MSC apoptosis, can inhibit macrophage pyroptosis and regulate the death process by decreasing the level of oxidative stress.

**Supplementary Information:**

The online version contains supplementary material available at 10.1186/s13287-023-03490-6.

## Introduction

Diabetes mellitus (DM) is a chronic metabolic condition that affects one in 11 individuals worldwide. Type 2 diabetes mellitus (T2DM) is the most common type of DM, comprising approximately 90% of cases [1]. Delayed healing of cutaneous wounds is a main complication of T2DM, and is difficult to treat [2, 3]. Apoptotic extracellular vesicles (ApoEVs) of mesenchymal stem cells (MSCs) are extracellular vesicles produced by apoptotic MSCs that have the ability to induce tissue regeneration. Our previous research revealed that MSC-derived ApoEVs improve cutaneous wound healing in normal C57BL/6 mice by improving fibroblast migration and proliferation abilities [4]. However, it remains unknown whether ApoEVs can promote the healing of skin lesions in patients with DM.

In chronic diabetic wounds, monocytes and macrophages accumulate and interleukin (IL)-1β production rises [5, 6]. In the case of NLRP3 (NOD-, LRR-, and pyrin domain-containing protein 3), the persistent inflammasome activity of wound macrophages also contributes to diabetic wound healing impairment [7–10]. These findings suggest that macrophage pyroptosis induced by activation of the NLRP3 cascade may play a critical role in delayed wound healing in patients with T2DM compared to normal wound healing.

Interestingly, an increasing number of studies have revealed that there is a direct mutual regulation and dynamic balance between different forms of cell death [11–13]. Apoptosis is a form of programmed cell death associated with anti-inflammatory activity that attenuates the immune response. Alternatively, pyroptosis is a form of inflammatory-induced programmed cell death that exacerbates the inflammatory response [12]. Activation of apoptotic caspase (caspase-3/7) has been shown to deactivate the pyroptosis marker protein gasdermin D (GSDMD) [14, 15]. In addition, in the macrophages with pyroptotic tendencies, activation of caspase-1 redirects cell fate toward apoptosis in the absence of GSDMD [16]. However, there have been no reports claiming that different forms of death for different cell types may regulate each other.

Umbilical cord mesenchymal stem cells (UCMSCs) can be obtained without any invasive surgery and have a higher proliferation efficiency than bone marrow-derived MSCs, which can promote wound healing and tissue regeneration in the injury process [17–20]. Here, we provide evidence that ApoEVs derived from UCMSCs can improve skin wound healing in db/db mice by inhibiting macrophage pyroptosis. ApoEVs may carry ectonucleotidases, E3 ubiquitin ligases, and miRNAs which dramatically reduce the oxidative stress status of macrophages, block the assembly of the inflammasome NLRP3, and ultimately suppress the pyroptosis process. Our findings indicate that apoptosis may regulate the progression of pyroptosis by transmitting information through the generation of ApoEVs. This study provides a unique, safe, and effective therapeutic approach for delayed wound healing in patients with T2DM.

## Materials and methods

### Animals

Our study reporting adhered to the ARRIVE guidelines for the reporting of animal experiments. All procedures were approved by the Institutional Animal Care and Use Committee of the Fourth Military Medical University and adhered to the Guide for the Care and Use of Laboratory Animals published by the National Institutes of Health. BKS-db mice (Strain No. T002407) were purchased from GemPharmatech (Nanjing, China) and C57BL/6 mice were purchased from the Laboratory Animal Center of the Fourth Military Medical University. Eight-week-old male db/db and db/m mice were used to establish cutaneous wound healing models, and eight-week-old male wildtype C57BL/6 mice were used for cell isolation. The mice were housed in a pathogen-free environment (24 °C, 12 h light/dark cycle, 50% humidity) with free access to food and drink. Euthanasia was performed by exposure to CO_2_ and was applied to all experimental mice. The mice were exposed to an automated CO_2_ delivery system and gradually lost consciousness. The concentration of CO_2_ was increased to 100% and to ensure that the mice did not react to a pinching finger response or dystonia, CO_2_ was ventilated for 2 min to achieve euthanasia.

### Cell culture

UCMSCs were provided by the stem cell sample bank of Tangdu Hospital of the Fourth Military Medical University. Cells were cultured in alpha Minimum essential medium (*α*-MEM) (Gibco, USA) supplemented with 10% fetal bovine serum (FBS) (Gibco, USA), 2 mM L-glutamine, 100 U/mL penicillin, and 100 g/mL streptomycin (all from Invitrogen, USA) at 37 °C with 5% CO_2_. Every 3 days, the culture medium was replenished. Passage 3 to 6 cells were used for all experiments.

Bone marrow-derived macrophages (BMDMs) were isolated from hind limbs of C57BL/6 mice. Bone marrow cells were isolated and washed with erythrocyte lysis buffer (Beyotime, China). After centrifugation at 100×*g* for 5 min, the cell suspension was resuspended and seeded in high-glucose (25 mM) Dulbecco’s modified Eagle’s medium (DMEM) (Gibco, USA) supplemented with 10% FBS, 2 mM L-glutamine, 100 U/mL penicillin, and 100 g/mL streptomycin. Mature macrophages were cultured with recombinant mouse macrophage colony-stimulating factor (M-CSF) (R&D, USA) at 50 ng/mL for 7 days. After 7 days, macrophages were treated with 1 μg/mL lipopolysaccharide (LPS) (Sigma-Aldrich, USA) to stimulate the inflammatory response for 12 h. ApoEVs at concentrations of 5 μg/mL, 10 μg/mL, and 20 μg/mL were used to stimulate the experimental group for another 6 h (the original culture medium was changed in the control group), and pyroptosis was induced by high-glucose DMEM containing 4 mM adenosine triphosphate (ATP) for 30 min. Photographs were taken under light microscopy.

After induction of pyroptosis, BMDMs were fixed with 2.5% glutaraldehyde for 12 h and washed three times with phosphate buffered saline (PBS) for 20 min each. After the final dehydration in graded alcohols, hexamethyldisilazane was used for drying. The dried samples were sent to the Instrumentation Center of the State Key Laboratory of Oral Medicine at the Fourth Military Medical University for spraying and scanning electron microscope photography.

### Isolation and characterization of ApoEVs

ApoEVs were isolated by an optimized method. First, the UCMSCs were treated with American staurosporine (STS) (Cell Signaling Technology, USA) at a concentration of 0.5 μM for 10 h to induce apoptosis. The supernatants were collected and centrifuged at 1000×*g* for 10 min at 4 °C to remove cells and debris. The supernatant was then centrifuged at 16,000×*g* for 30 min at 4 °C and washed twice in PBS. The isolated vesicles were resuspended in 100 μL PBS and stored at − 80 °C for further investigation. ApoEV content was quantified using the bicinchoninic acid (BCA) assay (Tiangen, China) before use. Apoptosis-induced UCMSCs were detached with 0.25% trypsin and centrifuged at 100×*g*. The apoptotic state was detected by flow cytometry using an Annexin V-FITC Apoptosis Detection Kit (7Sea Biotech, China), according to the manufacturer’s instructions. The morphological characteristics of ApoEVs were observed by scanning electron microscopy (SEM), and the size distribution and zeta potential of ApoEVs were determined using a Zetasizer Nano ZSE (Malvern, UK) and a dynamic light scattering instrument (DLS). Immunofluorescence staining was used to detect the expression of Annexin V and C1q in ApoEVs. Annexin V staining was performed using the Annexin V-FITC Apoptosis Detection Kit (7Sea Biotech, China). Caspase-3 and cleaved caspase-3 protein expression levels in UCMSC and ApoEV were detected by immunoblotting. To examine macrophage phagocytosis function in vivo and in vitro, ApoEVs were labeled with PKH26 or PKH67 and their co-localization with labeled fluorescent F4/80 cells was examined by confocal microscopy. Primary antibodies used in this study included F4/80 (Abcam, ab6640, UK), C1q (Abcam, ab11861, UK), GAPDH (CWBio, CW0100, China), caspase-3 (CST, 9662s, USA), and cleaved caspase-3 (CST, 9961s, USA).

### Flow cytometry identification of cell phenotype

UCMSCs and BMDMs were isolated with 0.25% trypsin and resuspended in PBS containing 3% FBS for flow cytometry analysis of cell surface markers. UCMSCs were then incubated in the dark for 30 min with phycoerythrin (PE)-conjugated human anti-CD34 antibody (Biolegend, 343506, USA), anti-CD45 antibody (Biolegend, 304058, USA), anti-CD105 antibody (Biolegend, 800504, USA), anti-CD90 antibody (eBioscience, 12-0909-41, USA); allophycocyanin (APC)-conjugated human anti-CD14 antibody (eBioscience, 17-0141-81, USA), anti-CD19 antibody (Biolegend, 302212, USA); and fluorescein isothiocyanate (FITC)-conjugated human anti-CD73 antibody (Biolegend, 344015, USA) and anti-HLA-DR antibody (Biolegend, 307603, USA). BMDMs were incubated with APC-conjugated mouse anti-F4/80 antibody (Biolegend, 123116, USA) and anti-CD11b antibody (Biolegend, 101206, USA) in the dark for 30 min. Associated conjugated immunoglobulins, provided by eBioscience and Biolegend, USA, were used as negative controls. Finally, cells were washed twice in PBS, and flow cytometry was used to detect positive cells (Beckman Coulter, USA).

### Establishment of a cutaneous wound healing model in T2DM mice

Eight-week-old male db/db and db/m mice were used to establish skin wound healing models (*n* = 7). Mice were anesthetized with pentobarbital sodium (40 mg/kg) via intraperitoneal injection prior to modeling. After shaving and cleaning the dorsal side of the mice, a 1-cm-diameter full-thickness wound was produced on the back skin. Fourteen db/db mice were randomly separated into two groups: the db/db group and the db/db + ApoEV group. Seven db/m mice served as healthy controls. Commercially available hydrogel Pluronic F-127 (PF-127) (Sigma-Aldrich, USA) was dissolved in PBS at a concentration of 30% at 4 °C. The UCMSC-derived ApoEVs were then embedded in the PF-127 solution at 4 °C, and the amount of vesicles applied to the wounds of the group db/db + ApoEVs was 50 μg (equivalent to 100 μL PF-127 solution), as assessed by the BCA assay. Equal volumes of blank gel were applied to the db/db and db/m groups, and the wounds were covered with American 3M surgical dressing after surgery. Mice were fed separately. The gel was applied on days 0 and 3, and the dressing was placed on the wound until the next time point. The wound area, body weight, and blood glucose levels were measured at 0, 3, 7, 10, and 14 days after surgery, and the wound healing rate was calculated according to the formula: Dn wound healing rate = (D0 wound area − Dn wound area)/D0 wound area × 100%. On day 14, mice were euthanized, and skin tissues were collected for further study.

### Histological staining

Skin tissue samples were fixed for 24 h in 4% paraformaldehyde and dehydrated with graded ethanol. The samples were then embedded in paraffin and sectioned to a thickness of 3 μm. Hematoxylin and eosin (H&E) staining and Masson staining were conducted with commercially available kits (Baso Technology, China). Digital images were obtained on a slice Pannoramic MIDI scanner (3DHISTECH, Hungary).

### Immunofluorescence staining

Cells and skin tissue samples were fixed overnight in 4% paraformaldehyde. Skin tissue samples were dehydrated with 30% sucrose, embedded in optimal cutting temperature (OCT) compound (Lecca, Germany), and sliced into 5 μm-thick sections. Cells and sections were permeabilized with 0.05% Triton X-100 (Sigma-Aldrich, USA) at room temperature for 10 min, blocked with 5% bovine serum albumin (Sigma-Aldrich, USA) for 30 min at 37 °C, and then incubated with primary antibodies overnight at 4 °C. The next day, the sections were incubated with fluorescent secondary antibodies (Sigma-Aldrich, USA) for 1 h at room temperature. Finally, they were left for 10 min at room temperature with DAPI (Sigma-Aldrich, USA). Images of cells were acquired by confocal microscopy, and images of slices were scanned using a Pannoramic MIDI scanner (3DHISTECH, Hungary). F4/80 and NLRP3, cleaved caspase-1, and GSDMD double-positive cells were counted using cell images. The intensity of immunofluorescence was assessed both in cell and in slice pictures. Primary antibodies involved in this study included F4/80 (Abcam, ab6640, UK), NLRP3 (Abcam, ab270449, UK), cleaved caspase-1 (CST, 89332s, USA), and GSDMD (Abcam, ab209845, UK).

### ELISA

After 3 days of establishing the skin wound model, the entire skin wounded area and the surrounding 5 mm full-thickness skin tissues were excised from the mice in each group, and were then homogenized in normal saline and centrifuged at 1000×*g*. The supernatants were collected for ELISA detection of IL-1β and IL-18 levels. In vitro cultured BMDMs were stimulated with LPS/ATP and different doses of ApoEVs, and the supernatant was collected and centrifuged at 1000×*g* after final induction. The supernatant was collected for IL-1β and IL-18 ELISA. IL-1β and IL-18 concentrations were detected by mouse ELISA kit (Neobioscience, China) according to the manufacturer’s instructions.

### Western blotting

For animal tissues, after 3 days of modeling the skin wound, the wounded skin area and surrounding 5 mm skin tissues were removed from the skin, homogenized in RIPA buffer containing protease inhibitors (Beyotime, China), and proteins were extracted. For BMDMs cultivated in high-glucose DMEM in vitro, cells at the bottom of the dish and dead cells in the supernatant were collected after stimulation with LPS/ATP and different concentrations of ApoEVs. The proteins were extracted with RIPA buffer containing protease inhibitors (Beyotime, China). All proteins were loaded onto sodium dodecyl sulfate–polyacrylamide (SDS) gels and were transferred to polyvinylidene fluoride (PVDF) membranes following BCA quantification (Milipoll, USA). The membranes were blocked with 5% bovine serum albumin for 2 h at room temperature, treated overnight at 4 °C with primary antibodies, and then incubated for 2 h at room temperature with peroxidase-conjugated secondary antibodies (CWBio, China). Protein bands were detected with an imaging system (Tanon, China) and quantified with Image J software. The main antibodies included β-actin (CWBio, CW0096, China), GAPDH (CWBio, CW0100, China), NLRP3 (Abcam, ab270449, UK), caspase-1 (Abcam, ab138483, UK), cleaved caspase-1 (CST, 89332s, USA), and GSDMD (Abcam, ab209845, UK).

### Lactate dehydrogenase (LDH) assay

BMDMs cultured in high-glucose DMEM in vitro were stimulated with LPS/ATP and different concentrations of ApoEVs. The supernatant was collected after the final induction, centrifuged at 1000×*g* to remove dead cells and cell debris, and the final supernatant was collected and detected with the LDH cytotoxicity assay kit (Nanjing Jiancheng, A020-2, China), according to the manufacturer’s instructions.

### Reactive oxygen species (ROS) detection

After establishing the skin wound in model mice, we treated the wound area with 50 μg ApoEVs (100 μL PF-127 solution) for 3 days. The skin wound area and the surrounding 5 mm-wide full-thickness skin tissue was removed from each group of mice, and homogenized in type I collagenase (2.5 mg/mL), which was then filtered through a 100-μm filter to obtain the cell suspension. All kinds of cells that can pass through the filter were collected for subsequent F4/80 and ROS fluorescence labeling and flow cytometry identification. The suspension was centrifuged at 100×*g* to obtain the cell pellet. For in vitro assays, cultured BMDM, after induction with LPS/ATP and 10 μg/mL ApoEVs as described above, cells were detached using 0.25% trypsin and centrifuged at 100×*g* to obtain cell pellets. Cell pellets were stained with F4/80-APC (Biolegend, 123116, USA) and ROS-FITC fluorescence (50101ES01, Yeasen, China) using a staining working solution according to the protocol provided by the manufacturer, washed twice with PBS, and positive rates and fluorescence intensities of F4/80 and FITC were detected by flow cytometry.

### Detection of enzymes and products related to oxidative stress

For mouse skin tissues, after 3 days of induction of the skin wound, the wounded area and surrounding 5 mm full-thickness skin tissue sections were excised from the mice in each group and homogenized in the extraction buffer or lysis buffer provided by the kit. The supernatant was retrieved for the detection of enzymes and products related to oxidative stress according to the kit instructions. For in vitro cultured BMDM cells, after induction by LPS/ATP and different concentrations of ApoEVs, 0.25% trypsin was used to detach cells. The dead cells in the supernatant and the cells at the bottom of the culture plate were collected and combined for lysis in extraction buffer or lysis buffer. Reagents were added according to the kit instructions. Oxidative stress enzymes and products were detected by a microplate reader using the following kits: Catalase (CAT) (KTB1040), Reduced Glutathione (GSH) (KTB1600), Micro Lipid Peroxidation Malondialdehyde (MDA) (KTB1050), and Micro Superoxide Dismutases (SOD) (KTB1030), all from abbkine.

### Phagocytosis blocking

BMDMs were cultured in vitro under the same conditions as above and stimulated with 0.25 mM Methyl Palmitate (MP) (T2S0157, Topscience, China) for 24 h after LPS stimulation to block the phagocytosis of macrophages, while ApoEVs were added afterward. Finally, pyroptosis was activated by ATP. Immunofluorescence was used to identify the reduction in ApoEV phagocytosis. Cells and culture medium were collected for Western blotting and ELISA identification.

For animal experiment, 1 h before the establishment of cutaneous wound healing model, MP (300 mg/kg) was injected intravenously, and the other modeling steps were the same as before. The phagocytosis reduction of ApoEVs was detected by Immunofluorescence. After 3 days of modeling, samples were obtained and sectioned. Then, immunofluorescence staining was performed to analyze the expression of NLRP3, cleaved capase-1, and GSDMD in F4/80-positive cells as before.

### Statistical analysis

In this study, all data are presented as mean ± standard deviation (SD). Two-tailed Student’s t tests were used to compare two groups. One-way analysis of variance (ANOVA) with Tukey correction was used to compare multiple groups and two-way ANOVA with Tukey correction was used to analyze the wound healing process. All statistical analyses were performed using GraphPad Prism 9.0 (GraphPad Software, USA). A *P* value < 0.05 was considered statistically significant.

## Results

### UCMSC-derived ApoEVs ameliorated delayed healing of cutaneous wounds in db/db mice

**Fig. 1:**
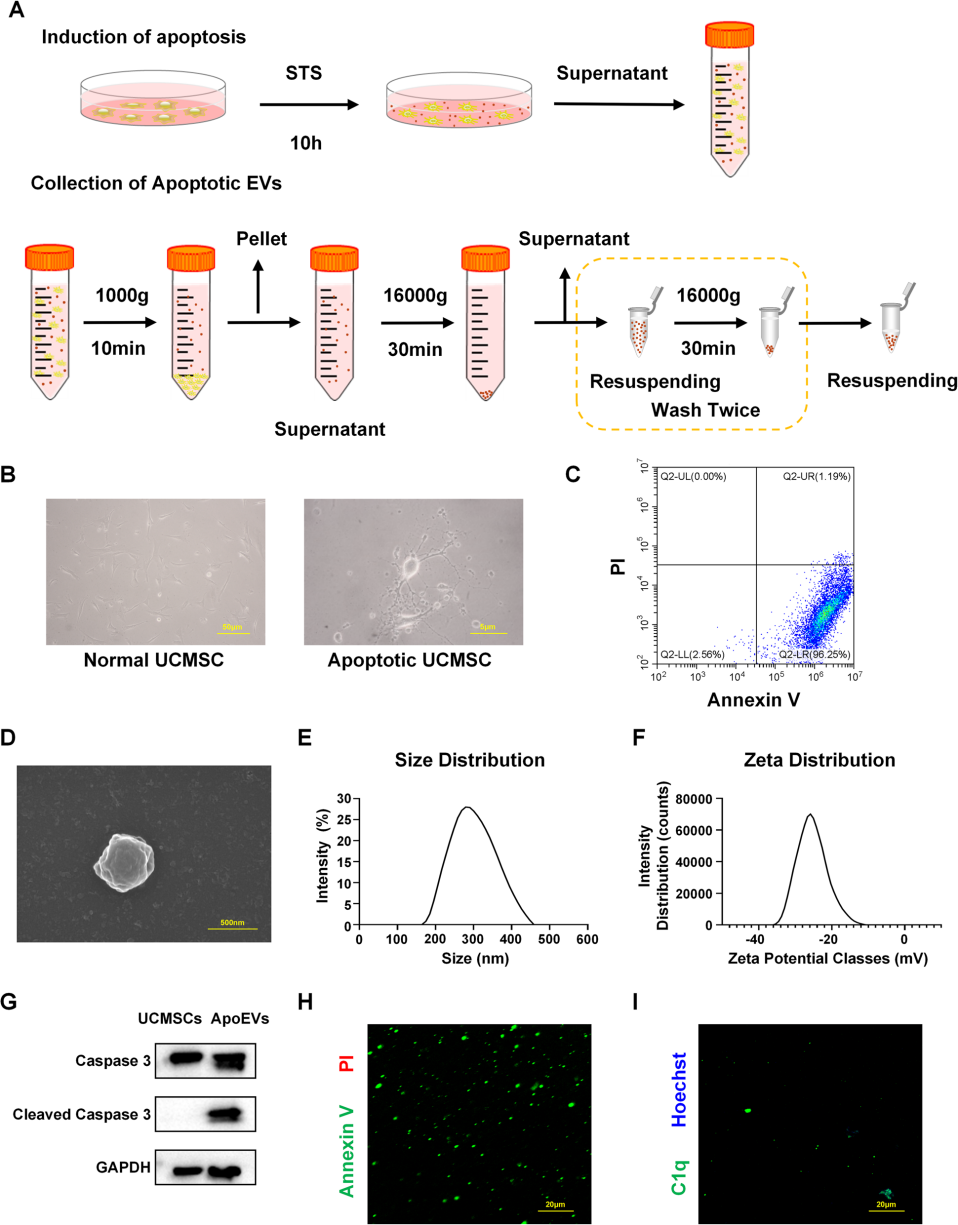
Isolation and characterization of UCMSC-derived ApoEVs. **a** Schematic graph of protocol for isolation of UCMSC-derived ApoEVs. **b** Cell morphology of normal and apoptotic UCMSCs. Scale bar, 50 μm in the left image, 5 μm in the right image. **c** Annexin V—PI expression of apoptotic UCMSCs analyzed by flow cytometer. **d** Representative image of SEM analysis of UCMSC-derived ApoEVs. Scale bar, 500 nm. **e, f** Size and zeta potential distribution of UCMSC-derived ApoEVs measured by DLS. **g** The protein level of caspase-3 and cleaved caspase-3 for UCMSC-derived ApoEVs examined by western blots. Corresponding uncropped full-length gels and blots are presented in Additional file 5A. **h, i** Representative images of Annexin V and C1q staining of UCMSC-derived ApoEVs. Scale bar, 20 μm

UCMSCs at P3 were used for surface marker identification by flow cytometry (Additional file 1A). Next, UCMSC apoptosis was induced with STS and ApoEVs were isolated by a gradient centrifugation protocol (Fig. 1A) for future characterization and utilization. Mature UCMSCs were polygonal or fusiform, whereas apoptotic UCMSCs were elongated with beaded pseudopodia at the edges (Fig. 1B). Annexin V-PI was utilized to identify apoptotic UCMSCs by flow cytometry. The results revealed that 96.25% apoptotic cells were in the early phase of the process (Fig. 1C). ApoEVs were identified by measuring their size distribution and zeta potential using SEM and DLS analysis (Fig. 1D–F). The particle size of ApoEVs used in this study was around 300 nm, and their zeta potential was − 25 V. Using western blotting, we also detected caspase-3 and cleaved caspase-3 expression in UCMSCs and their ApoEVs (Fig. 1G). Immunofluorescence labeling was also employed to determine the expression of the apoptotic markers Annexin V and C1q in ApoEVs (Fig. 1H, I).

**Fig. 2:**
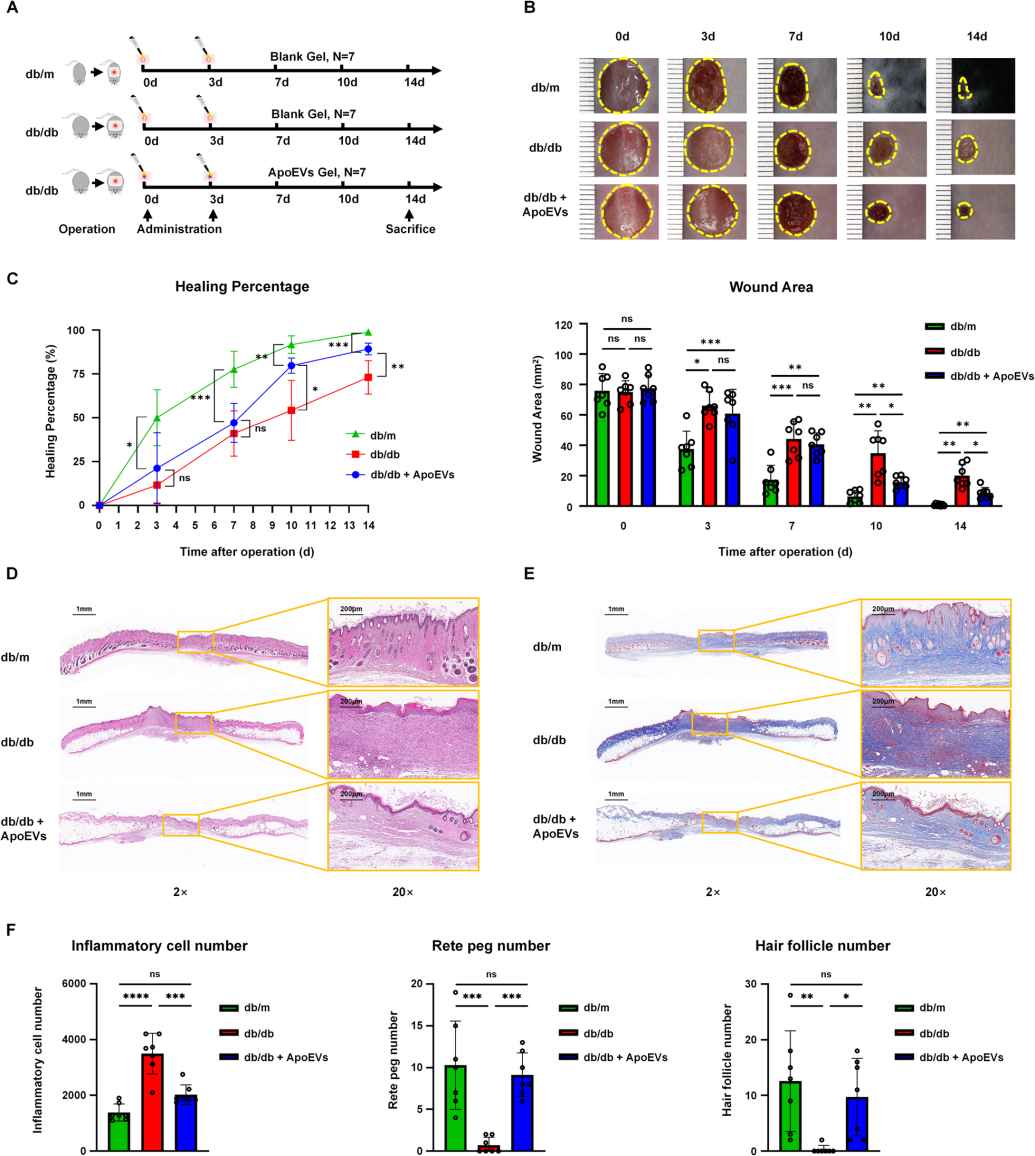
UCMSC-derived ApoEVs ameliorated cutaneous wound healing in db/db mice. **a** The protocol of establishment and treatment for mouse cutaneous wound models, *N* = 7 per group. **b** Representative photographs of cutaneous wounds in different groups at different time points during the wound healing process. **c** Quantification of the wound healing percentage and wound area. **d** Representative images of the H&E staining of the skin samples. Scale bar, 1 mm in low-magnification images, 200 μm in high-magnification images. **e** Representative images of the Masson staining of the skin samples. Scale bar, 1 mm in low-magnification images, 200 μm in high-magnification images. **f** Quantification of inflammatory cell number, rete peg number, and hair follicle number. *P < 0.05; **P < 0.01; ***P < 0.001; ****P < 0.0001; *NS* not significant. Blank Gel, PF-127 Gel; ApoEVs Gel, UCMSC-derived ApoEVs embedded in PF-127 Gel

To evaluate the therapeutic efficacy of UCMSC-derived ApoEVs, we created a cutaneous defect in db/db mice and utilized db/m mice as a healthy control (Fig. 2A). Among the three groups, db/m mice healed the quickest and reached a peak healing rate at 3 days after surgery; however, db/db mice exhibited a delayed wound healing phenomenon and a reduced wound healing rate. As anticipated, the wound healing state of the db/db ApoEVs group improved dramatically, the wound area was significantly reduced and the healing rate was statistically significantly greater than that of the db/db group 10 days after trauma. However, ApoEVs treatment did not bring the healing rate and minimal wound area to those of the level in normal mice (Fig. 2B, C). During this process, we also discovered that db/db mice had considerably higher body weight and blood glucose levels than db/m mice; ApoEVs had no influence on the body weight and blood glucose levels of mice (Additional file 2A, B).

On day 14, we obtained skin samples and performed histological analysis to evaluate the outcome of skin regeneration. H&E staining revealed that the scar sites of db/db mice had a greater number of inflammatory cells than db/m mice. In the db/db group, we also observed a lack of hair follicle regeneration and an inadequate skin structure. ApoEV-treated db/db mice had lower inflammatory cell infiltration, more intact skin structure with rete pegs, and more specific hair follicle production than db/db mice (Fig. 2D, F Additional file 4A). However, the skin appendages of ApoEV-treated db/db mice were not as intact as those of db/m mice, and the skin fat content was higher in db/db mice due to T2DM (Additional file 2C). Furthermore, the results of Masson staining also showed that the db/db ApoEVs treatment group had a better organized collagen structure than the db/db group (Fig. 2E, Additional file 4B). By examining H&E-stained organ slides, we discovered no significant histological alterations between the organs of mice in each group (Additional file 2D), indicating that local application of ApoEVs is safe.

Through in vivo imaging, we also observed the systemic spread of vesicles after local administration. After 72 h, the vesicles remained mostly distributed around the wound area and did not accumulate significantly in other tissues and organs (Additional file 2E).

### UCMSC-derived ApoEVs inhibited macrophage pyroptosis during skin wound healing in db/db mice

**Fig. 3:**
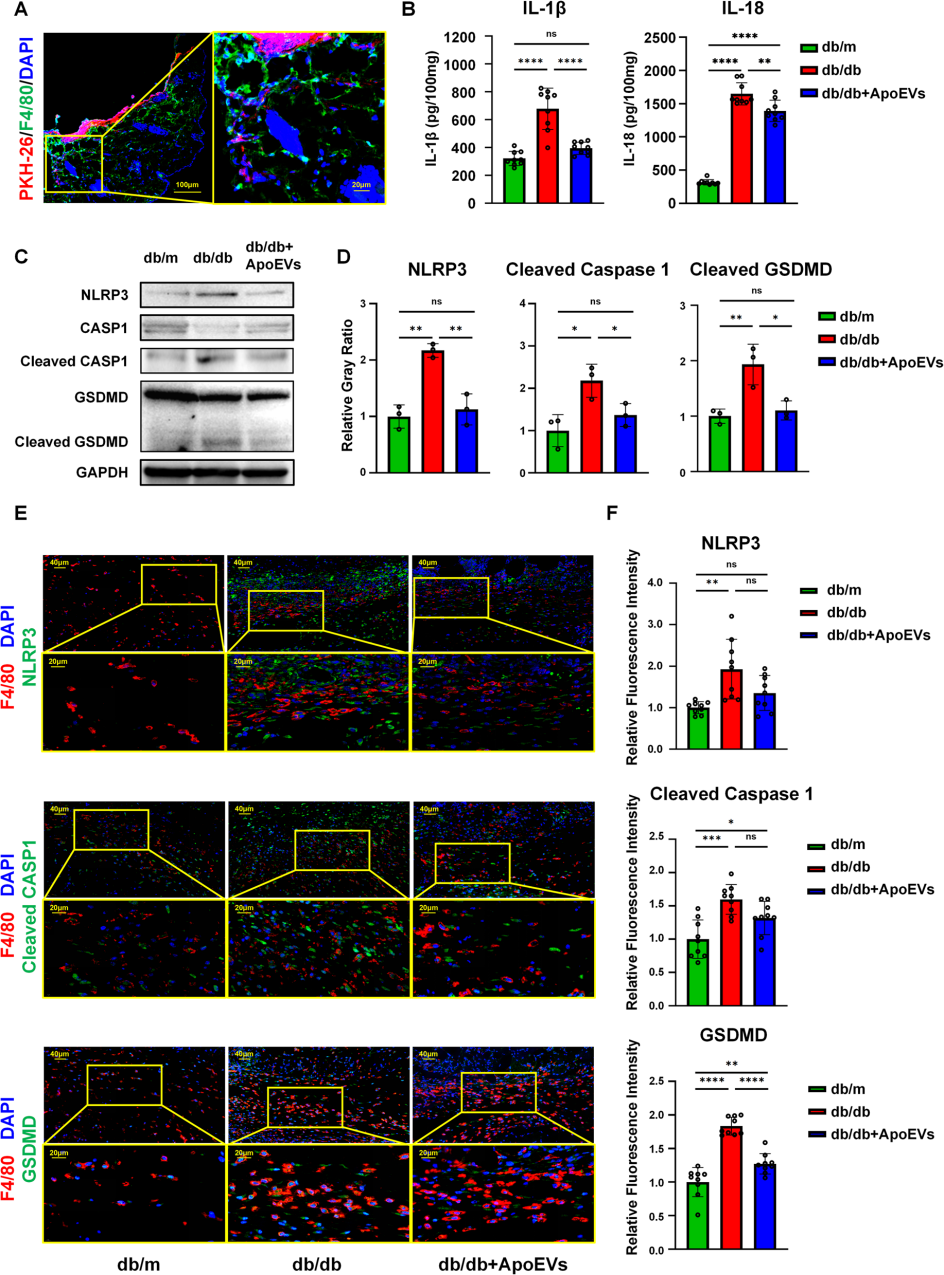
UCMSC-derived ApoEVs inhibited macrophage pyroptosis present during skin defect healing in db/db mice. **a** Immunofluorescence staining shows UCMSC-derived ApoEVs phagocytosed by F4/80-positive macrophages in vivo. Scale bar, 100 μm in low-magnification images, 20 μm in high-magnification images. **b** Tissue inflammatory factors IL-1β and IL-18 of different groups released in and around mouse skin defect area detected by ELISA, *N* = 3 per group, 3 duplication per sample. **c** Tissue protein levels of NLRP3, caspase-1, cleaved caspase-1, gasdermin D, cleaved gasdermin D of different groups released in and around mouse skin defect area detected by Western Blots, *N* = 3 per group. Corresponding uncropped full-length gels and blots are presented in Additional file 5B. **d** Semi-quantification of protein expression of NLRP3, cleaved caspase-1, and cleaved gasdermin D. **e** Immunofluorescence staining shows NLRP3, cleaved caspase-1, and gasdermin D in the skin defect area of mice in all three groups had significant co-localization with F4/80-positive macrophages. Scale bar, 40 μm in low-magnification images, 20 μm in high-magnification images. *N* = 3 per group, 3 ROIs per sample. **f** Semi-quantification of NLRP3, cleaved caspase-1, and gasdermin D relative fluorescence intensity in F4/80-positive cells of skin defect area. Data shown as mean ± SD. *P < 0.05; **P < 0.01; ***P < 0.001; ****P < 0.0001; *NS* not significant. *CASP1* caspase-1; *GSDMD*, gasdermin D

In the experiments described above, we discovered that the local application of ApoEVs improved healing rather than acting through the systemic system. As a result, we focused on macrophages at the wound site and labeled ApoEVs with PKH26 to see if they could be phagocytosed and then affect macrophages. Following local application, ApoEVs were largely phagocytosed by F4/80-positive macrophages (Fig. 3A).

The contents of IL-1β and IL-18 in the local wound tissue of db/db mice with T2DM were significantly higher than those of healthy db/m mice, according to ELISA using the supernatant of skin tissue homogenates. When ApoEVs were applied topically to db/db mice wounds, the levels of IL-1β and IL-18 were significantly reduced (Fig. 3B), indicating that ApoEVs exerted a significant anti-inflammatory effect and inhibited pyroptosis. After homogenizing tissues, the expression of pyroptosis-related proteins was identified by western blotting; the results showed that the expression of NLRP3 inflammasomes, activated cleaved caspase-1, and cleaved GSDMD-N in the db/db group increased significantly compared to the db/m group, whereas ApoEVs could significantly lower the expression of pyroptosis proteins (Fig. 3C, D).

The co-localization of proteins related to pyroptosis and the macrophage surface marker F4/80 in the skin defect was determined by immunofluorescence labeling and slide scanning. F4/80-positive macrophages significantly co-localized with pyroptosis-related proteins, such as NLRP3, cleaved caspase-1, and GSDMD, in the skin wound area in all three groups. Additionally, the db/db group displayed considerably higher fluorescence intensities of pyroptosis-related proteins than those of the db/m group. While in the db/db ApoEVs group, these were somewhat down-regulated (Fig. 3E, F).

These results suggested that during the repair of skin wounds in db/db mice, substantial numbers of macrophages located in the wounded area underwent pyroptosis, while ApoEVs can inhibit the upstream of pyroptosis by reducing NLRP3 inflammasome production.

### UCMSC-derived ApoEVs inhibited LPS/ATP-induced macrophage pyroptosis in a high-glucose environment

**Fig. 4:**
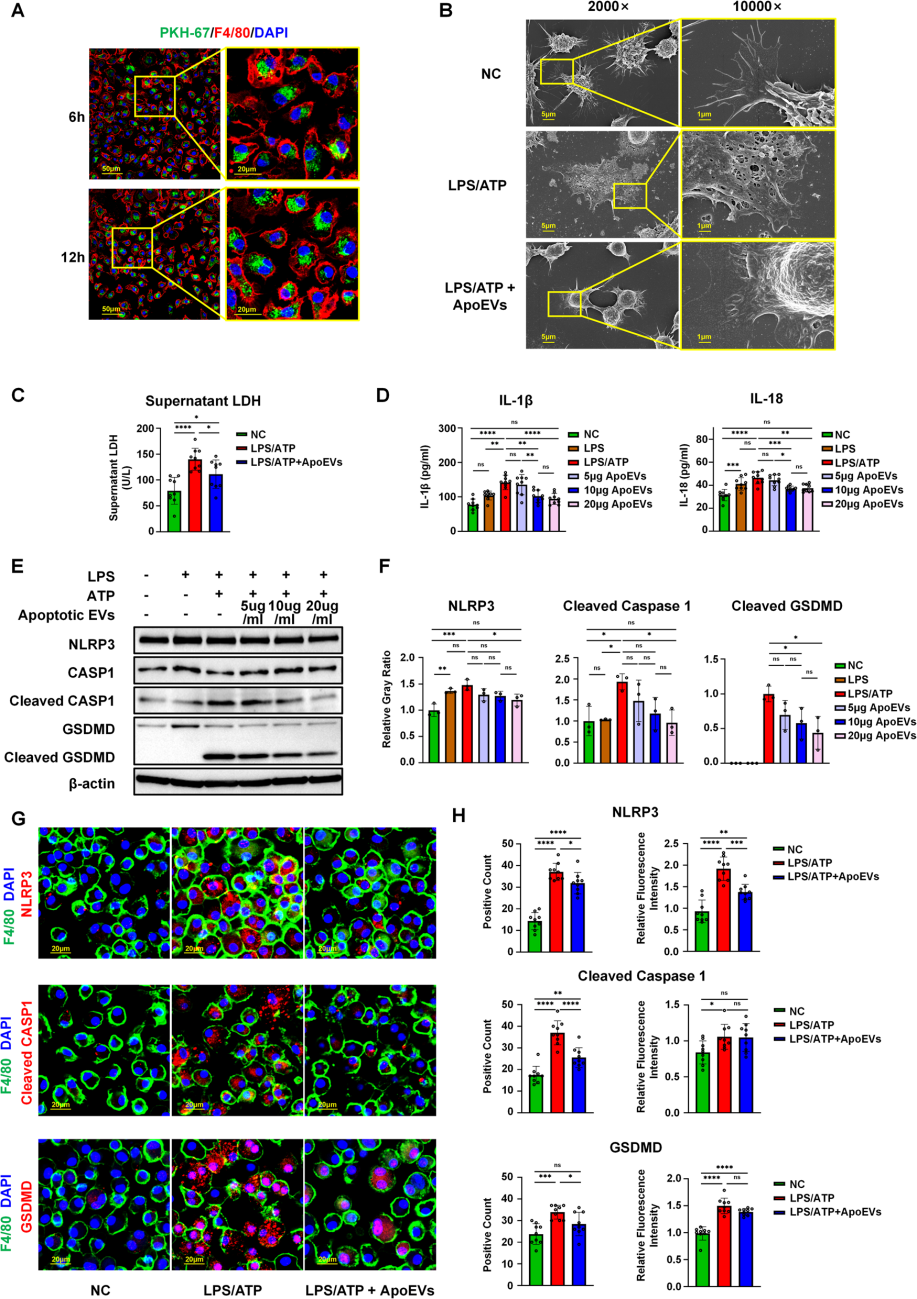
UCMSC-derived ApoEVs inhibited LPS/ATP-induced macrophage pyroptosis in a high-glucose environment. **a** Immunofluorescence staining shows UCMSC-derived ApoEVs phagocytosed by F4/80-positive macrophages in vitro. Scale bar, 50 μm in low-magnification images, 20 μm in high-magnification images. **b** Representative image of SEM analysis of macrophages treated with LPS/ATP and UCMSC-derived ApoEVs. Scale bar, 5 μm in low-magnification images, 1 μm in high-magnification images. **c** Quantification of supernatant LDH under different stimulation conditions, *N* = 3 per group, 3 duplication per sample. **d** The detection of inflammatory factors IL-1β and IL-18 released by macrophages under different stimulation conditions by ELISA, *N* = 3 per group, 3 duplication per sample. **e** The protein levels of NLRP3, caspase-1, cleaved caspase-1, gasdermin D, cleaved gasdermin D of macrophages treated with different concentrations of UCMSC-derived ApoEVs examined by Western Blots, *N* = 3 per group. Corresponding uncropped full-length gels and blots are presented in Additional file 5C. **f** Semi-quantification of protein expression of NLRP3, cleaved caspase-1, and cleaved gasdermin D. **g** Immunofluorescence staining shows NLRP3, cleaved caspase-1, and gasdermin D had significant co-localization with F4/80-positive macrophages in vitro. Scale bar, 20 μm. **h** Semi-quantification of NLRP3, cleaved caspase-1, and gasdermin D positive count and relative fluorescence intensity. *N* = 3 per group, 3 ROIs per sample. Data shown as mean ± SD. *P < 0.05; **P < 0.01; ***P < 0.001; ****P < 0.0001; *NS* not significant. *LDH* lactate dehydrogenase; *CASP1* caspase-1; *GSDMD* gasdermin D

To verify that ApoEVs could ameliorate macrophage pyroptosis in a high-glucose environment in vitro, we cultured BMDMs from C57BL/6 mice under high-glucose conditions. After 7 days, mature BMDMs were used for flow cytometric phenotyping (Additional file 1B). We next labeled ApoEVs with PKH67 fluorescent dye and co-cultured them with macrophages. ApoEVs were able to be phagocytosed by the majority of F4/80-positive macrophages 6 h after co-culture, but the number of positive cells did not increase appreciably after 12 h (Fig. 4A).

Mature BMDMs were stimulated with LPS/ATP for acute inflammation and induction of pyroptosis to imitate acute trauma, and UCMSC-derived ApoEVs were then used to intervene during stimulation. Under SEM (Fig. 4B), normal BMDMs had smooth surface protrusions with pseudopodia extending around, whereas LPS/ATP-induced BMDMs flattened and the cell body was covered with round or oval holes with pseudopodia extending only slightly. The BMDMs in the ApoEVs-treated group presented a more stereoscopic morphology than the LPS/ATP group, with pseudopodia extending around and only tiny foramina appearing at particular positions of the cell body. Morphological photographs of BMDMs were also obtained under light microscopy (Additional file 1C). For further confirmation, we determined the degree of cell lysis and death by measuring LDH levels in the generated supernatant. Following induction of LPS/ATP, concentration of LDH in the supernatant was dramatically elevated. LDH was reduced in the supernatant of the ApoEV-treated group, while it remained higher than that in the control group (Fig. 4C).

Cell supernatants were collected for ELISA detection of pyroptosis-related proteins after induction. In addition, cells were harvested and proteins were isolated for Western blotting to determine the expression level of pyroptosis-related proteins. The results of ELISA showed that ApoEVs could significantly suppress the increase of IL-1β and IL-18 in BMDMs induced by LPS/ATP under a high-glucose environment (Fig. 4D). Western blotting results revealed that, in hyper-glucose cultured BMDMs, LPS/ATP dramatically up-regulated the expression of NLRP3 and cleaved caspase-1, creating a cleaved GSDMD-N band and inducing pyroptosis. In contrast, ApoEVs significantly reduced the expression of NLRP3, cleaved caspase-1, and GSDMD-N in a dose-dependent manner (Fig. 4E, F). Immunofluorescence staining was also performed to test macrophage pyroptosis in vitro. The results demonstrated that NLRP3, cleaved caspase-1, GSDMD, and F4/80 were co-localized in pyroptotic BMDMs, and that the proportion of positive cells and the relative fluorescence intensity were significantly higher in the LPS/ATP-induced group than in the control group, which could be reduced by ApoEVs (Fig. 4G, H).

Therefore, we demonstrated that UCMSC-derived ApoEVs could significantly inhibit the assembly of the NLRP3 inflammasome in BMDMs induced by LPS/ATP in a high-glucose environment, which then inhibited pyroptosis. These results were comparable with those of the in vivo experiments obtained using db/db mice.

### UCMSC-derived ApoEVs exerted its effect through being phagocytized by macrophages

To verify that UCMSC-derived ApoEVs exert its effect through being phagocytosed by macrophages, rather than through membrane contact and other ways to inhibit pyroptosis, supplementary experiments of ApoEV function test after using MP were performed.

**Fig. 5:**
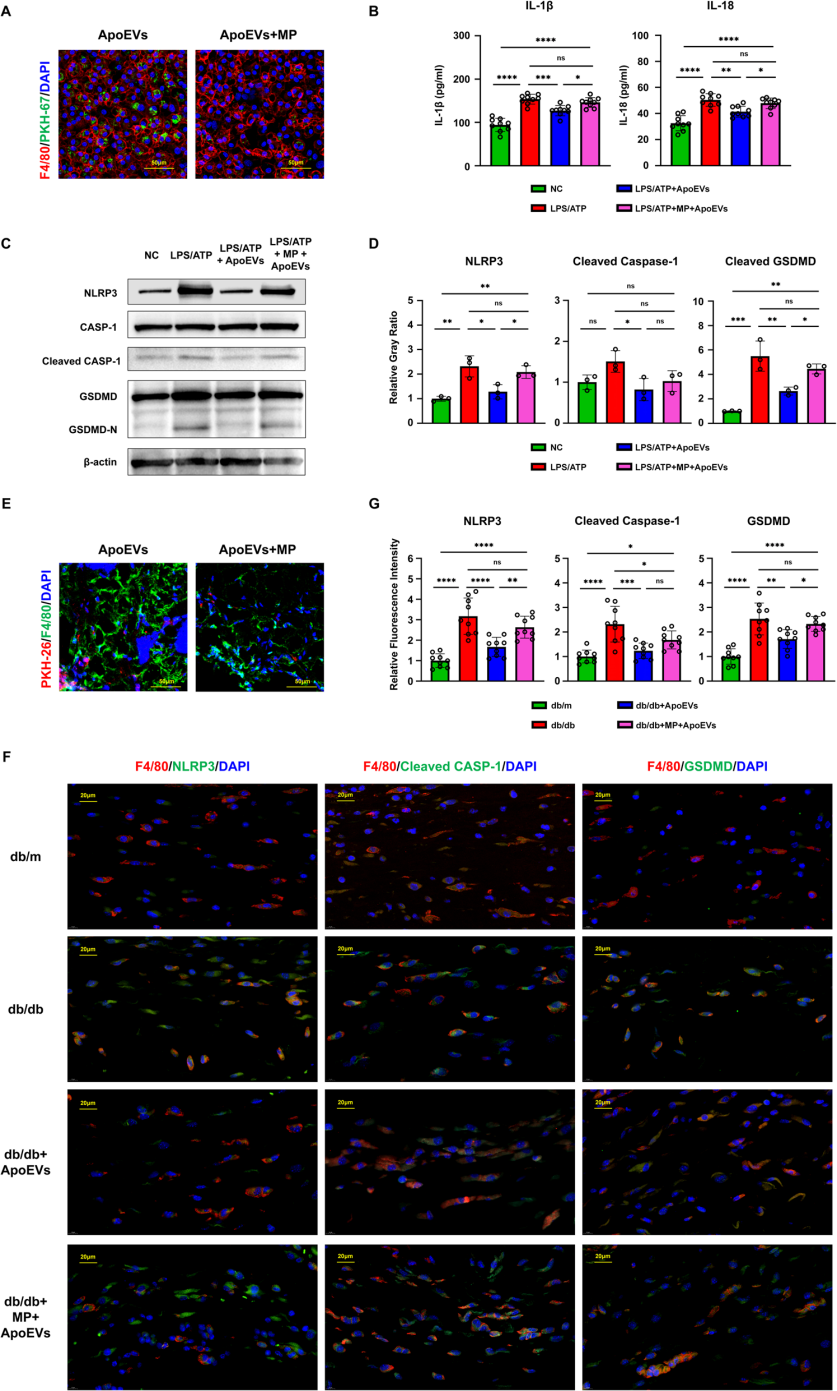
The function of ApoEVs was impaired after using methyl palmitate. **a** Immunofluorescence staining shows that the function of BMDMs to phagocytize ApoEVs is inhibited in vitro. Scale bar, 50 μm. **b** The detection of inflammatory factors IL-1β and IL-18 released by BMDMs under different stimulation conditions by ELISA, *N* = 3 per group, 3 duplication per sample. **c** The protein levels of NLRP3, caspase-1, cleaved caspase-1, gasdermin D, cleaved gasdermin D of BMDMs treated with LPS/ATP, ApoEVs, and methyl palmitate, *N* = 3 per group. Corresponding uncropped full-length gels and blots are presented in Additional file 5D. **d** Semi-quantification of protein expression of NLRP3, cleaved caspase-1, and cleaved gasdermin D. **e** Immunofluorescence staining shows that the function of macrophages to phagocytize ApoEVs is inhibited in vivo. Scale bar, 50 μm. **f** Immunofluorescence staining in vivo shows the expression of NLRP3, cleaved caspase-1, and gasdermin D in F4/80-positive macrophages changed with the intervention of methyl palmitate. Scale bar, 20 μm. *N* = 3 per group, 3 ROIs per sample. **g** Semi-quantification of NLRP3, cleaved caspase-1, and gasdermin D positive count and relative fluorescence intensity. *N* = 3 per group. Data shown as mean ± SD. *P < 0.05; **P < 0.01; ***P < 0.001; ****P < 0.0001; NS, not significant. MP, methyl palmitate; CASP1, caspase-1; GSDMD, gasdermin D

In vitro, immunofluorescence staining shows that the function of BMDMs to phagocytize ApoEVs is inhibited by MP (Fig. 5A). The results of ELISA showed that both IL-1β and IL-18 levels produced by pyroptosis could be reduced by ApoEVs, and the use of MP resulted in a significant upregulation (Fig. 5B). Western blotting results also showed that ApoEVs significantly reduced the expression of pyroptosis proteins, and if ApoEVs were used after the administration of MP, the pyroptosis-protein expression would increase again (Fig. 5C, D).

In vivo, immunofluorescence staining also shows that the macrophage function to phagocytize ApoEVs is inhibited by MP (Fig. 5E). For slices, the fluorescence intensities of pyroptosis-related proteins in the db/m,db/db,db/db ApoEVs group acted as before. While after the intervention of MP, the pyroptosis inhibition function of ApoEVs reduced and the fluorescence intensities of pyroptosis-related proteins raised again (Fig. 5F, G).

### UCMSC-derived ApoEVs ameliorated the state of oxidative stress in macrophages

Studies have shown that elevated glucose levels can form excessive ROS in the process of oxidative phosphorylation of glucose and produce oxidative stress in macrophages [21]. In addition, both human and animal investigations have demonstrated that oxidative stress-related enzymes and metabolites play a significant role in poor wound healing [22–26]. Furthermore, ROS may serve as a trigger to activate NLRP3 inflammasomes, leading to pyroptosis [27]. Therefore, we speculated that the delayed skin wound healing in db/db mice is most likely due to ROS accumulation and macrophage pyroptosis resulting from enhanced oxidative stress, whereas ApoEVs can effectively inhibit ROS accumulation, reduce the oxidative stress status of macrophages, and subsequently prevent the occurrence of pyroptosis.

**Fig. 6:**
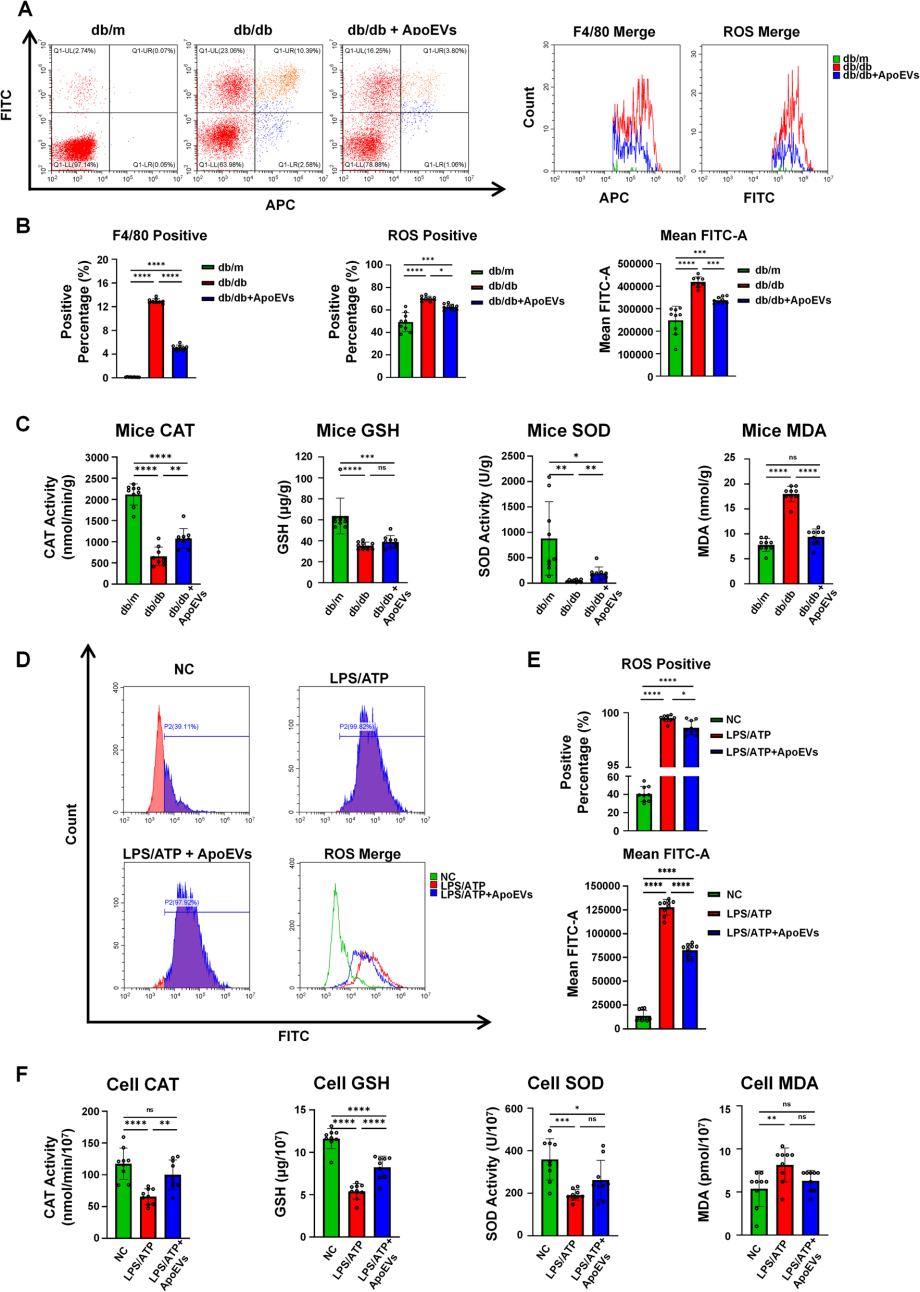
UCMSC-derived ApoEVs ameliorated oxidative stress status in macrophages (*N* = 3 for in vivo experiments). **a** F4/80 and ROS expression of collagenase isolated tissue cells in and around mouse skin defect area analyzed by flow cytometer, *N* = 3 per group, 3 duplication per sample. **b** Quantification of F4/80-positive percentage of all isolated cells, ROS positive percentage, and fluorescence intensity of F4/80-positive cells. **c** Quantification of CAT, GSH, SOD, and MDA of tissue in and around mouse skin defect area, *N* = 3 per group, 3 duplication per sample. **d** ROS expression of macrophages under different stimulation conditions analyzed by flow cytometer, *N* = 3 per group, 3 duplication per sample. **e** Quantification of ROS positive percentage and fluorescence intensity of macrophages under different stimulation conditions. **f** Quantification of CAT, GSH, SOD, and MDA of macrophages under different stimulation conditions, *N* = 3 per group, 3 duplication per sample. Data shown as mean ± SD. *P < 0.05; **P < 0.01; ***P < 0.001; ****P < 0.0001; *NS* not significant. *ROS* reactive oxygen species; *CAT* catalase; *GSH* glutathione; *SOD* superoxide dismutase; *MDA* malondialdehyde

In vivo, tissue cells were isolated from the skin on the dorsal side of the mouse surrounding the wound (including the wound itself) by type I collagenase and were fluorescently labeled for both ROS and F4/80 following assessment by flow cytometry. ApoEVs could effectively suppress the recruitment of F4/80-positive macrophages in the vicinity of the skin defect in db/db mice. The ROS fluorescence intensity and ROS positive proportion in F4/80-positive macrophages of db/db mice were considerably higher than those of the db/m group but decreased in the ApoEVs group (Fig. 6A, B).

In addition, the local tissue of the skin defect was homogenized, and the local oxidative stress level was determined. In comparison with normal db/m mice, the levels of CAT, GSH, and SOD were much lower in db/db mice, while the level of MDA was significantly higher. However, in mice treated with db/db ApoEVs, CAT, GSH, and SOD levels increased, whereas MDA levels dropped, although the change in GSH was not statistically significant (Fig. 6C). These findings indicated that ApoEVs could considerably reduce oxidative stress in db/db mice during wound healing, which may be a key mechanism by which ApoEVs prevent macrophage pyroptosis.

In vitro, the ROS fluorescence intensity and positive proportion of BMDMs were measured by flow cytometry before and after ApoEVs treatment. The levels of oxidative stress in macrophages were also measured. The results showed that after LPS/ATP stimulation, the proportion of ROS positive cells and fluorescence intensity in BMDMs were significantly increased, and this aberrant growth was partially reduced by ApoEVs (Fig. 6D, E). Similarly, CAT, GSH, and SOD levels were significantly down-regulated, and MDA was significantly up-regulated after LPS/ATP stimulation. ApoEVs were able to partially restore CAT, GSH, and SOD levels and reduce MDA generation (Fig. 6F), which was consistent with the findings in the animal experiments.

**Fig. 7:**
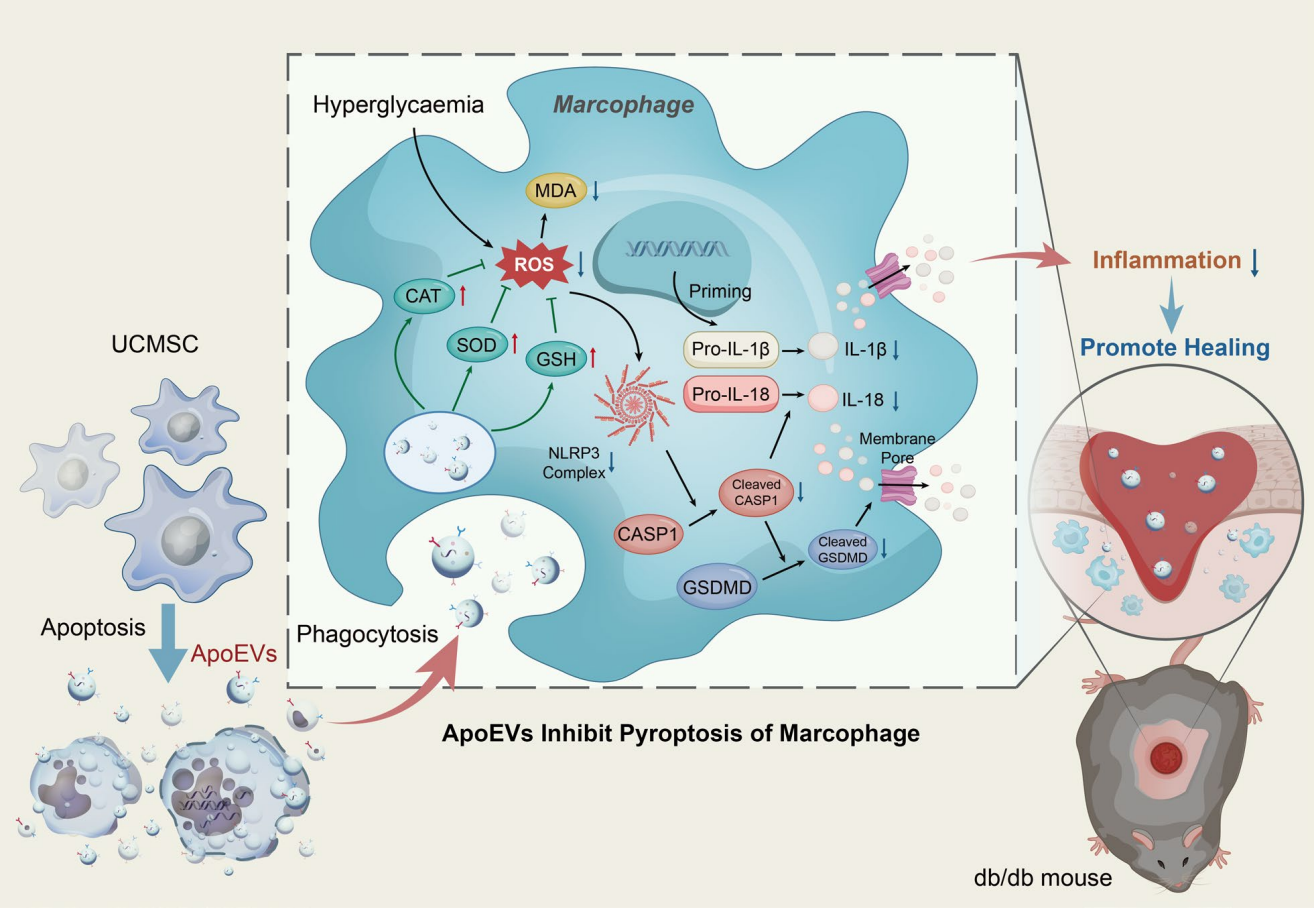
Schema indicates that UCMSC-derived ApoEVs, after being phagocytosed by macrophages, exert their anti-inflammation efficacy by relieving oxidative stress status, reducing the production and assembly of the inflammasome NLRP3, and then inhibiting macrophage pyroptosis, thus provide a promising therapy for delayed cutaneous wound healing of T2DM. *ROS* reactive oxygen species; *CAT* catalase; *GSH* glutathione; *SOD* superoxide dismutase; *MDA* malondialdehyde; *IL-1β* interleukin-1 beta; *IL-18* interleukin-18; *CASP1* caspase-1; *GSDMD* gasdermin D

The above experimental results indicated that aggravation of the oxidative stress level in macrophages during wound healing in db/db mice was an important factor causing pyroptosis, triggering strong inflammation, and ultimately leading to delayed wound healing, while UCMSC-derived ApoEVs could significantly reduce the oxidative stress level of macrophages, inhibit pyroptosis, and finally improve the wound healing effect (Fig. 7).

## Discussion

T2DM has a high incidence worldwide and is commonly accompanied by poor wound healing, which is difficult to treat and has gained a wide focus by researchers [1–3]. Macrophages are recruited around diabetic wounds with inflammasome activation and undergo pyroptosis, triggering increased inflammation [5–9]. This constitutes an important reason for the delayed healing of diabetic skin defects, which differs from the normal healing process. The phenomenon of massive transplanted MSCs undergoing apoptosis and production of ApoEVs has been observed in various therapeutic models for MSCs, including skin wounds in healthy mice, and researchers have further confirmed that ApoEVs derived from MSCs play an essential role in tissue regeneration [4, 28–31]. However, it is unclear whether ApoEVs can also promote tissue regeneration to improve delayed healing due to differences between diabetic and normal wounds. In addition, researchers have been highly concerned about the dynamic balance of different forms of cell death. For one type of cell, apoptosis and pyroptosis can regulate or even transform into each other under certain conditions [11–13]. For example, there have been studies in cancer attempting to transform apoptosis into pyroptosis in order to eradicate tumor cells [32, 33]. However, no study has shown that different forms of death for different cell types could regulate each other. Our study used ApoEVs produced by apoptotic UCMSCs as media to inhibit macrophage pyroptosis, which subsequently improved cutaneous wound healing in T2DM mice. Meanwhile, the administration of ApoEVs was able to regulate different forms of cell death in different cell types. This suggests a novel therapeutic approach for T2DM-delayed wound healing and provides a new understanding of the mutual regulation of cell death. Unfortunately, there is no conclusive evidence that apoptosis can prevent pyroptosis. It is difficult to confirm whether there is such a regulatory effect in the case of physiological conditions without man-made intervention, particularly due to the difficulty of directly tracing extracellular vesicles under physiological conditions. This requires additional investigation in future, but our research undoubtedly confirmed apoptotic products could influence pyroptosis, shedding a light on the study of cell death regulation.

In vivo, we established cutaneous wound healing models in BKS-db mice and used ApoEVs to rescue abnormal healing in the db/db group. Consistent with Van Tuan Nguyen's research, the healing of skin wounds in db/db mice was substantially slower than in db/m mice [3]. However, when ApoEVs were applied topically, diabetic wounds began to recover significantly faster than untreated wounds. We also confirmed that T2DM mice had higher expression of pyroptosis-related proteins, such as NLRP3, and increased expression of inflammatory factors, such as IL-1β, than normal mice, which is consistent with Rita Mirza and Alessandra Bitto’s studies [7, 8, 34]. Immunofluorescence scanning results presented that pyroptosis-related proteins had significant co-localization with F4/80-positive macrophages. Compared with normal mice, the fluorescence intensities of pyroptosis-related proteins were significantly higher in diabetic mice, but decreased following administration of ApoEVs. Indeed, altogether these investigations verified the conclusion of delayed wound healing in diabetic skin, as well as the presence of NLRP3 and elevated inflammatory factors, implying the role of pyroptosis in delayed wound healing, but there is no in-depth discussion of this in their research. Furthermore, other studies have clearly demonstrated the importance of macrophages in chronic wounds, including diabetic wounds [3, 35–37], but these studies on macrophages focused more on the phenotypic transformation of M1/M2, and generally ignored the effects of other macrophage behaviors. In contrast, our study used ApoEVs for the first time to treat the delayed healing of diabetic skin wounds. ApoEVs have also been shown to play a role in macrophage pyroptosis, which is a strong process that supports the importance of macrophages in healing.

In vitro, LPS/ATP was used to stimulate BMDMs cultured in a high-glucose environment to mimic macrophage pyroptosis in a diabetic environment. UCMSC-derived ApoEVs were also administered during the LPS/ATP induction. We found that LPS/ATP could significantly increase NLRP3 inflammasome activity and cause macrophage pyroptosis in BMDMs in a high-glucose environment, corroborating earlier research [38–40]. Through ApoEVs intervention, the pyroptotic state of BMDMs was dramatically reduced, as evidenced by enhanced stereoscopic morphology, improved membrane integrity, decreased production of pyroptosis-related proteins, downregulation of inflammatory markers, etc. There have previously been reports of extracellular vesicles preventing pyroptosis, such as Zhenzhen Hu's and Xiaoli Liu's studies [41, 42], but these studies used exosomes, and our study is the first to incorporate ApoEVs into pyroptosis-inhibiting vesicles. ApoEVs are distinct from other extracellular vesicles in that they are the product of apoptosis rather than the typical state of living cells.

Additionally, we utilized MP to block macrophage phagocytosis of ApoEVs. ApoEVs could not continue to perform a function when macrophage phagocytosis was suppressed, according to the findings. The pyroptosis level of BMDMs was not reduced after LPS/ATP stimulation, and inflammatory factors were not down-regulated as ApoEVs group. However, these results of palmitate-treated group seemed not to achieve the pyroptosis level of the LPS/ATP group (this may be because a small portion of the ApoEVs was still phagocytosed by BMDMs). It indicates that ApoEVs only play a role after being phagocytosed by macrophages, rather than through membrane contact and other ways to inhibit pyroptosis. Moreover, the results of immunofluorescence also confirmed the hypothesis.

In addition to defining a potential therapeutic application of ApoEVs, we also conducted some mechanistic research. Using ROS flow cytometry and oxidative stress kits such as GSH, CAT, SOD, and MDA, we confirmed that the oxidative stress levels of macrophages in db/db mice were significantly higher than those in normal mice, as previously reported for diabetic patients and animal models [22–26]. Local administration of ApoEVs can significantly alleviate oxidative stress. In vitro, we also detected macrophage oxidative stress levels after LPS/ATP-induced pyroptosis in a high-glucose environment. The results corresponded to those in vivo. The level of oxidative stress in BMDMs after pyroptosis induction was significantly increased, which was consistent with the study by Shen et al. on macrophages [43]. However, this response was greatly mitigated with the use of ApoEVs.

Our research has certain limitations. For example, the primary objective of our work was to evaluate the inflammatory response generated by the pyroptotic state of macrophages, whereas the functional cell states during wound healing were ignored. In fact, the effect of ApoEVs on skin fibroblasts has been illustrated in our previous study [4]. Additionally, this work did not provide a comprehensive understanding of the mechanism by which ApoEVs suppress pyroptosis; it merely demonstrated that ApoEVs relieved the oxidative stress state of macrophages. Furthermore, our study did not identify the vesicle contents that targeted different molecules in order to achieve its inhibitory activity, which necessitates further exploration in future studies. Nonetheless, based on the literature, we hypothesized that certain ApoEV components could be involved. As typical ectonucleotidases, CD39 and CD73 have been shown to alleviate oxidative stress in cells and can inactivate ATP that can eventually hydrolyze into adenosine, rendering further activation of NLRP3 ineffective [44]. In fact, MSCs overexpress these two proteins and release them through vesicles [45–47]; thus, we hypothesized that ApoEVs express high levels of CD39/CD73 and play a crucial role in the process of preventing pyroptosis. In addition, Liu et al. demonstrated that ApoEVs could enhance tissue regeneration via transferring an E3 ubiquitin ligase and miRNA [48]. However, the E3 ubiquitin ligases and miRNAs carried in UCMSCs-derived ApoEVs have not been identified, which also warrants additional investigation. In our future studies, we will investigate the functional components of ApoEVs.

Our previous study has demonstrated that systemic application of MSC-derived ApoEVs reduced T2DM features such as insulin resistance, but ApoEVs were mainly metabolized in the liver [49]. In this study, only topical application of gel containing ApoEVs was used, which drastically minimized the amount of vesicles utilized. The topical application avoided adverse effects on organ systems and was more conducive to improving the safety of treatment while saving costs. In addition, the fact that local administration can improve wound healing proved once more that macrophage pyroptosis plays an essential role in the wound healing process of T2DM. From this perspective, this study offers a novel therapeutic concept for the treatment of delayed diabetic wound healing. Conversely, this study also demonstrated, for the first time, that ApoEVs of UCMSCs can inhibit the pyroptosis state of macrophages. ApoEVs, unlike other extracellular vesicles, are produced by apoptosis and are not products of the normal state of living cells. This suggests that there may be an orderly regulatory process between different forms of death for different cell types, using extracellular vesicles as a mediator. This proposed mechanism should be further confirmed by future in-depth studies.

## Conclusions

The present study showed that UCMSC-derived ApoEVs significantly accelerated the delayed healing of skin lesions in db/db mice with natural T2DM. During this process, we also verified that UCMSC-derived ApoEVs exercise their efficacy by alleviating macrophage oxidative stress status, lowering NLRP3 inflammasome production, and then preventing macrophage pyroptosis (Fig. 7). This safe method of topical application provides a new approach for the treatment of delayed wound healing complications in T2DM. The result, ApoEVs inhibited pyroptosis, which also establishes a novel theoretical foundation for the mutual regulation of various forms of cell death.

### Supplementary Information

**Additional file 1**: Flow cytometric analysis and morphological photographs. **a** Flow cytometric analysis of the UCMSCs’ surface markers showed positive expression of CD73, CD90, and CD105, and negative expression of hematopoietic markers HLA-DR, CD14, CD19, CD34, and CD45. **b** Flow cytometric analysis of the BMDMs’ surface markers showed positive expression of F4/80 and CD11b. **c** Representative morphological photographs of macrophages treated with LPS/ATP and UCMSC-derived ApoEVs. Scale bar, 50μm.

**Additional file 2**: Local application of UCMSC-derived ApoEVs improved healing rather than acting through the systemic system. **a**, **b** Quantification of the body weight and blood glucose of mice, N=7 per group. **c** Representative images of the H&E staining of the skin samples before surgery. Scale bar, 200 μm. **d** Representative images of the H&E staining of organs of mice in each group. Scale bar, 1 mm. **e** Systemic distribution after local application of vesicles observed by small-animal in vivo imaging.

**Additional file 3**: Other photographs of cutaneous wounds not shown in the figure.

**Additional file 4**: Images of histological staining of other slices not shown in the figure. **a** Images of the H&E staining of the skin samples. Scale bar, 1 mm in low-magnification images, 100 μm in high-magnification images. **b** Images of the Masson staining of the skin samples. Scale bar, 1 mm in low-magnification images, 200 μm in high-magnification images.

**Additional file 5**: Corresponding uncropped full-length gels and blots. **a** Corresponding uncropped full-length gels and blots of Fig. 1G. **b** Corresponding uncropped full-length gels and blots of Fig. 3C. **c** Corresponding uncropped full-length gels and blots of Fig. 4E. **d** Corresponding uncropped full-length gels and blots of Fig. 5C. **e** Other corresponding uncropped full-length gels and blots of pyroptosis inhibition in vivo not shown in the figure. **f** Other corresponding uncropped full-length gels and blots of pyroptosis inhibition in vitro not shown in the figure. **g** Other corresponding uncropped full-length gels and blots of methyl palmitate intervention not shown in the figure.

## Data Availability

Not applicable.

## Abbreviations

DM: Diabetes mellitus
T2DM: Type 2 diabetes mellitus
ApoEVs: Apoptotic extracellular vesicles
MSCs: Mesenchymal stem cells
UCMSCs: Umbilical cord mesenchymal stem cells
IL: Interleukin
NLRP3: NOD-, LRR-, and pyrin domain-containing protein 3
GSDMD: Gasdermin D
CASP: Caspase
α-MEM: Alpha minimum essential medium
FBS: Fetal bovine serum
BMDMs: Bone marrow-derived macrophages
DMEM: Dulbecco’s modified Eagle’s medium
M-CSF: Macrophage colony-stimulating factor
LPS: Lipopolysaccharide
ATP: Adenosine triphosphate
PBS: Phosphate-buffered saline
STS: Staurosporine
BCA: Bicinchoninic acid assay
SEM: Scanning electron microscopy
DLS: Dynamic light scattering instrument
PF-127: Pluronic F-127
PE: Phycoerythrin
APC: Allophycocyanin
FITC: Fluorescein isothiocyanate
H&E: Hematoxylin and eosin staining
OCT: Optimal cutting temperature compound
SDS: Sodium dodecyl sulfate–polyacrylamide
PVDF: Polyvinylidene fluoride membranes
LDH: Lactate dehydrogenase
ROS: Reactive oxygen species
CAT: Catalase
GSH: Glutathione
MDA: Malondialdehyde
SOD: Superoxide dismutase
MP: Methyl palmitate
