# Supplementary figures and images for: Umbilical cord mesenchymal stem cell-derived apoptotic extracellular vesicles ameliorate cutaneous wound healing in type 2 diabetic mice via macrophage pyroptosis inhibition
Source: Stem Cell Res Ther. 2023 Sep 19;14:257. doi: 10.1186/s13287-023-03490-6 (PMC10510296; doi:10.1186/s13287-023-03490-6)

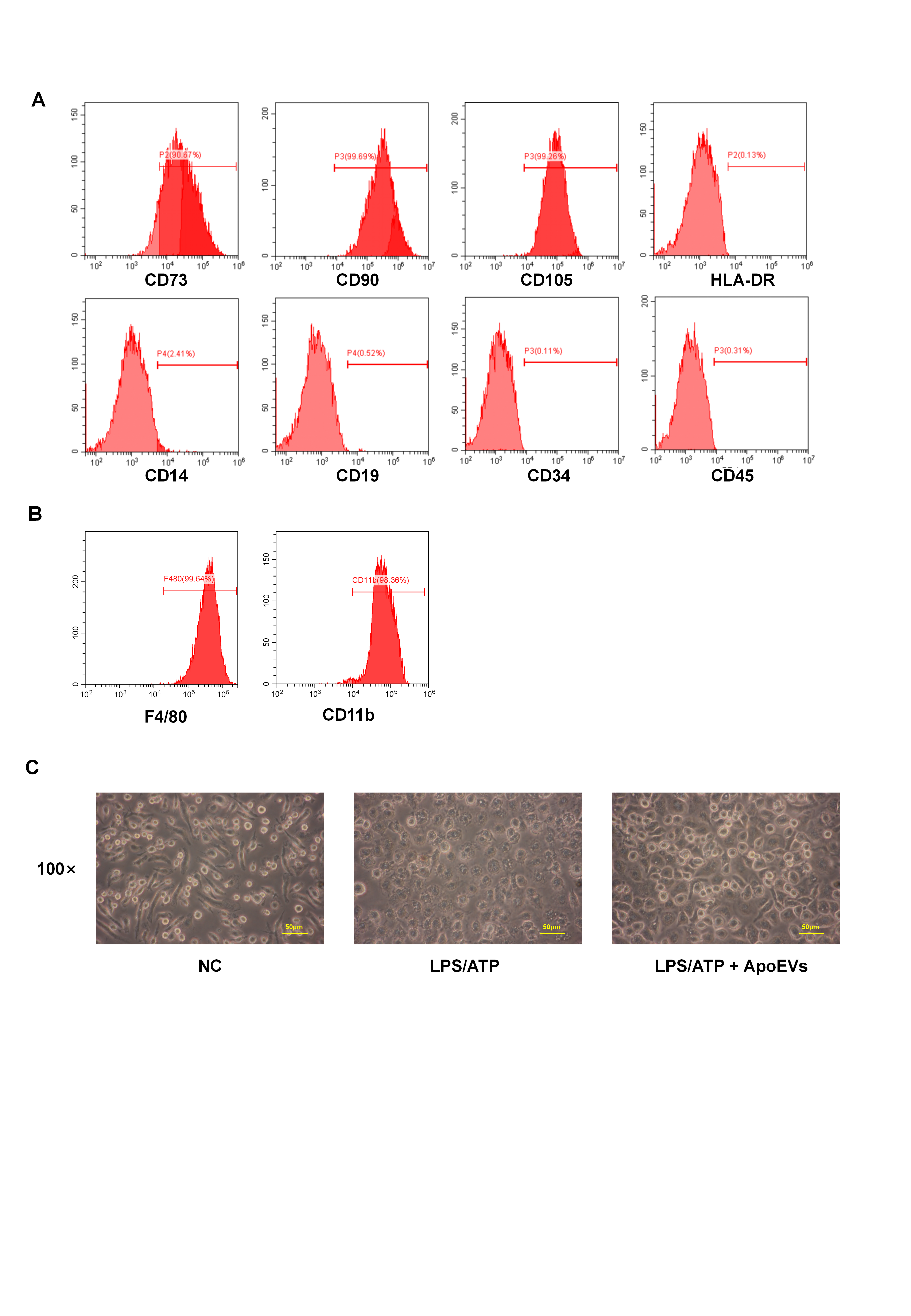

Supplement: Supplementary file 1 — Additional file 1: Flow cytometric analysis and morphological photographs. a Flow cytometric analysis of the UCMSCs’ surface markers showed positive expression of CD73, CD90, and CD105, and negative expression of hematopoietic markers HLA-DR, CD14, CD19, CD34, and CD45. b Flow cytometric analysis of the BMDMs’ surface markers showed positive expression of F4/80 and CD11b. c Representative morphological photographs of macrophages treated with LPS/ATP and UCMSC-derived ApoEVs. Scale bar, 50μm. [file 13287_2023_3490_MOESM1_ESM.png]

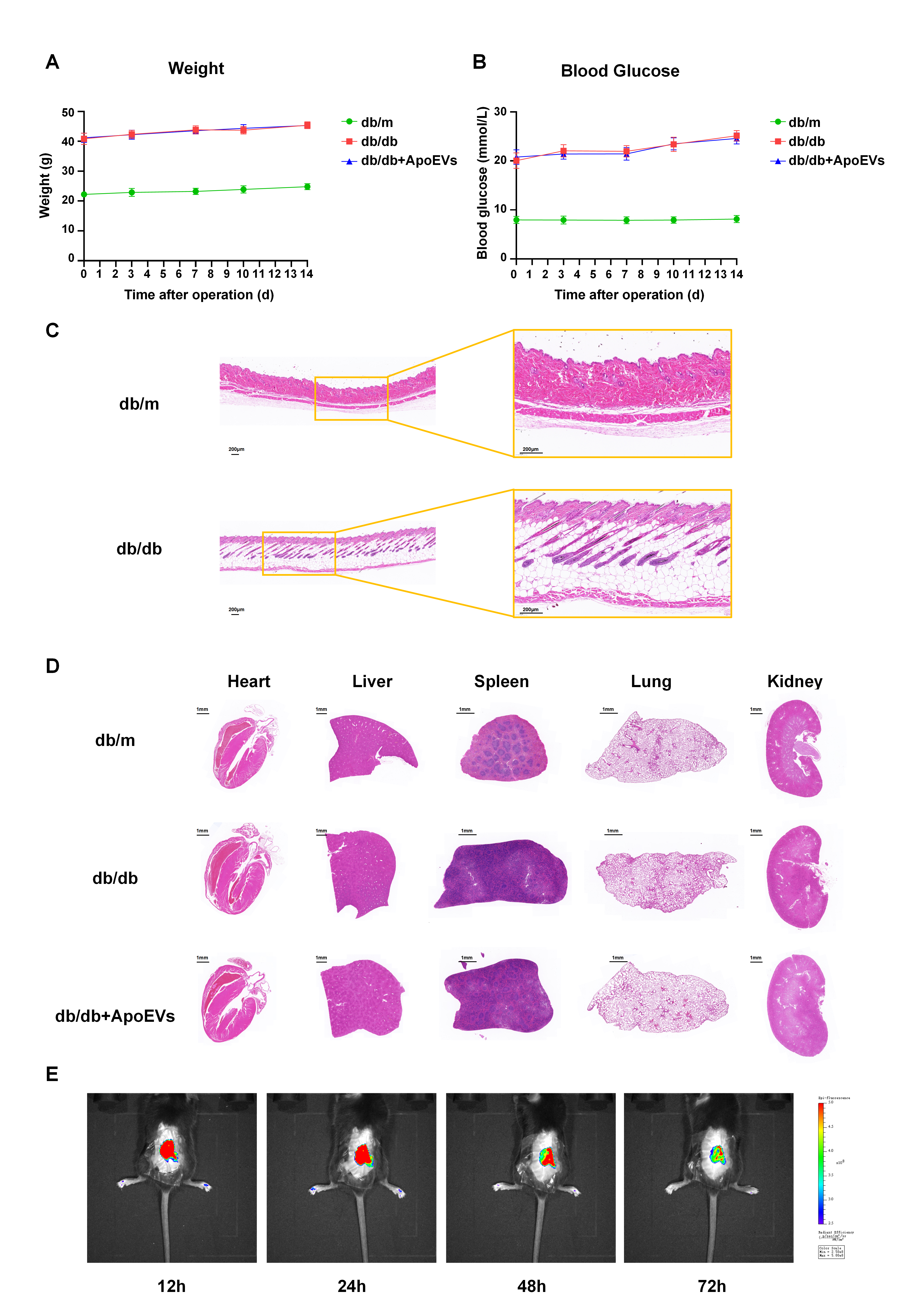

Supplement: Supplementary file 2 — Additional file 2: Local application of UCMSC-derived ApoEVs improved healing rather than acting through the systemic system. a, b Quantification of the body weight and blood glucose of mice, N=7 per group. c Representative images of the H&E staining of the skin samples before surgery. Scale bar, 200 μm. d Representative images of the H&E staining of organs of mice in each group. Scale bar, 1 mm. e Systemic distribution after local application of vesicles observed by small-animal in vivo imaging. [file 13287_2023_3490_MOESM2_ESM.png]

**Additional File 3**

**Other photographs of cutaneous wounds not shown in the figure.**

**
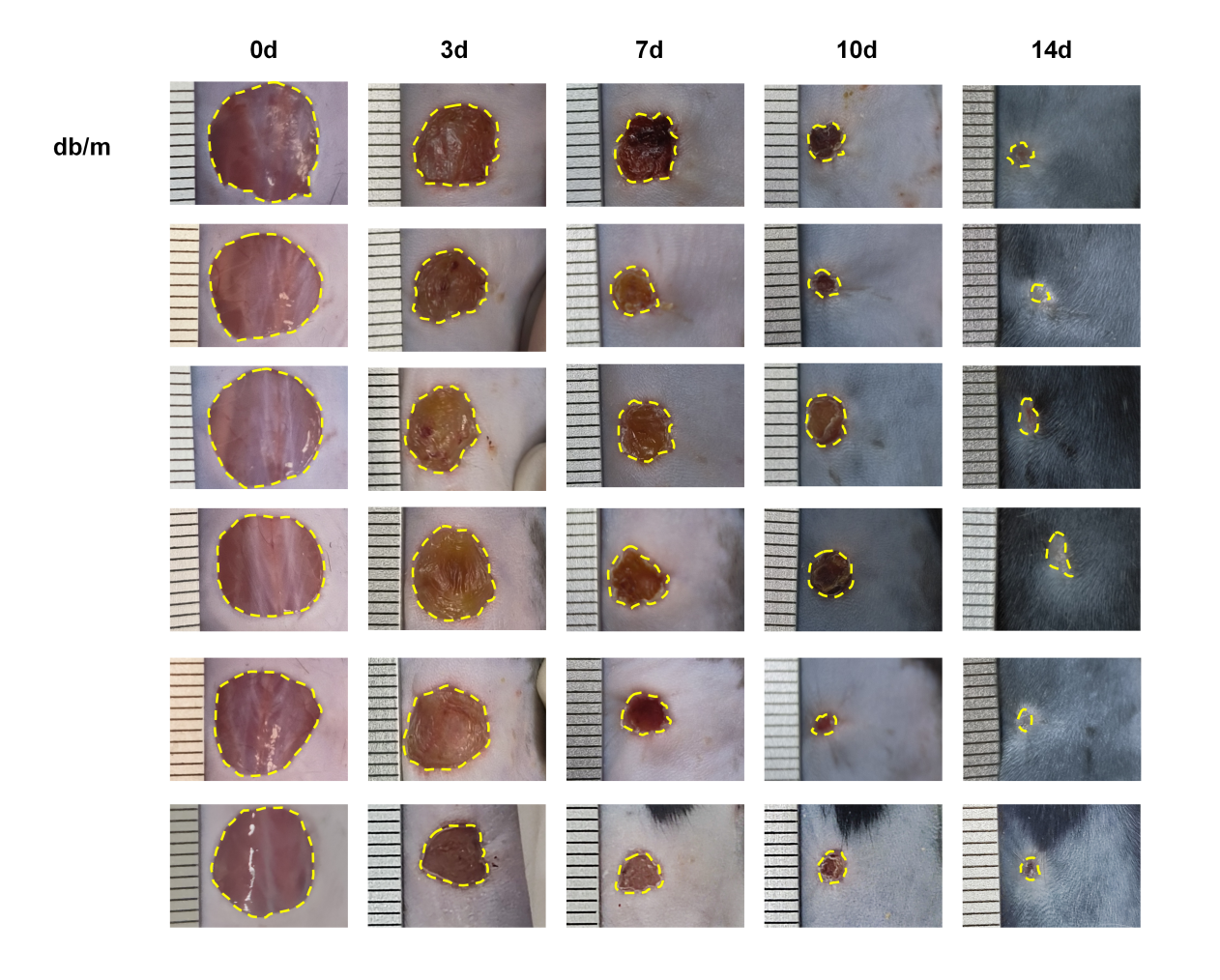
**

**
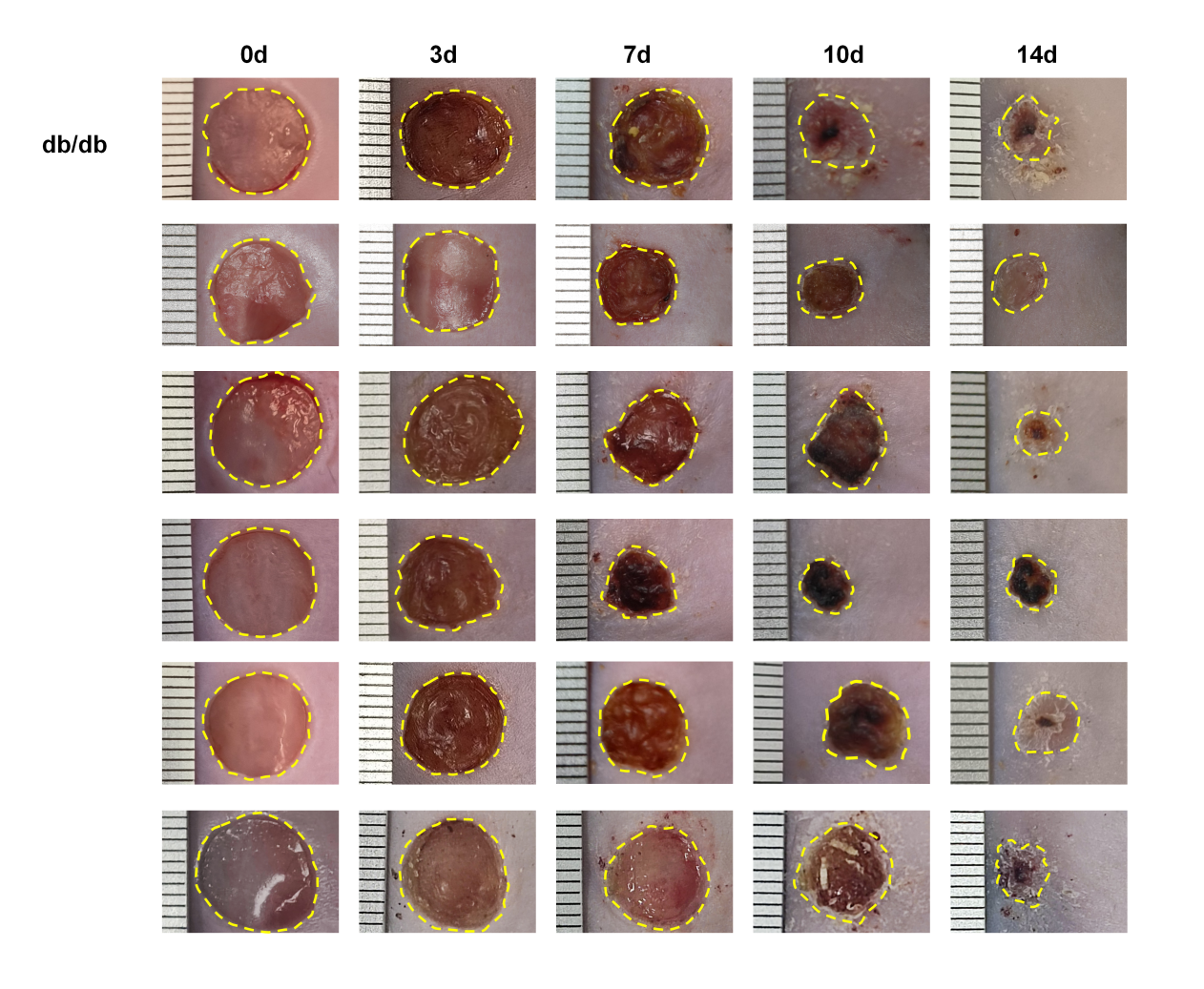
**

**
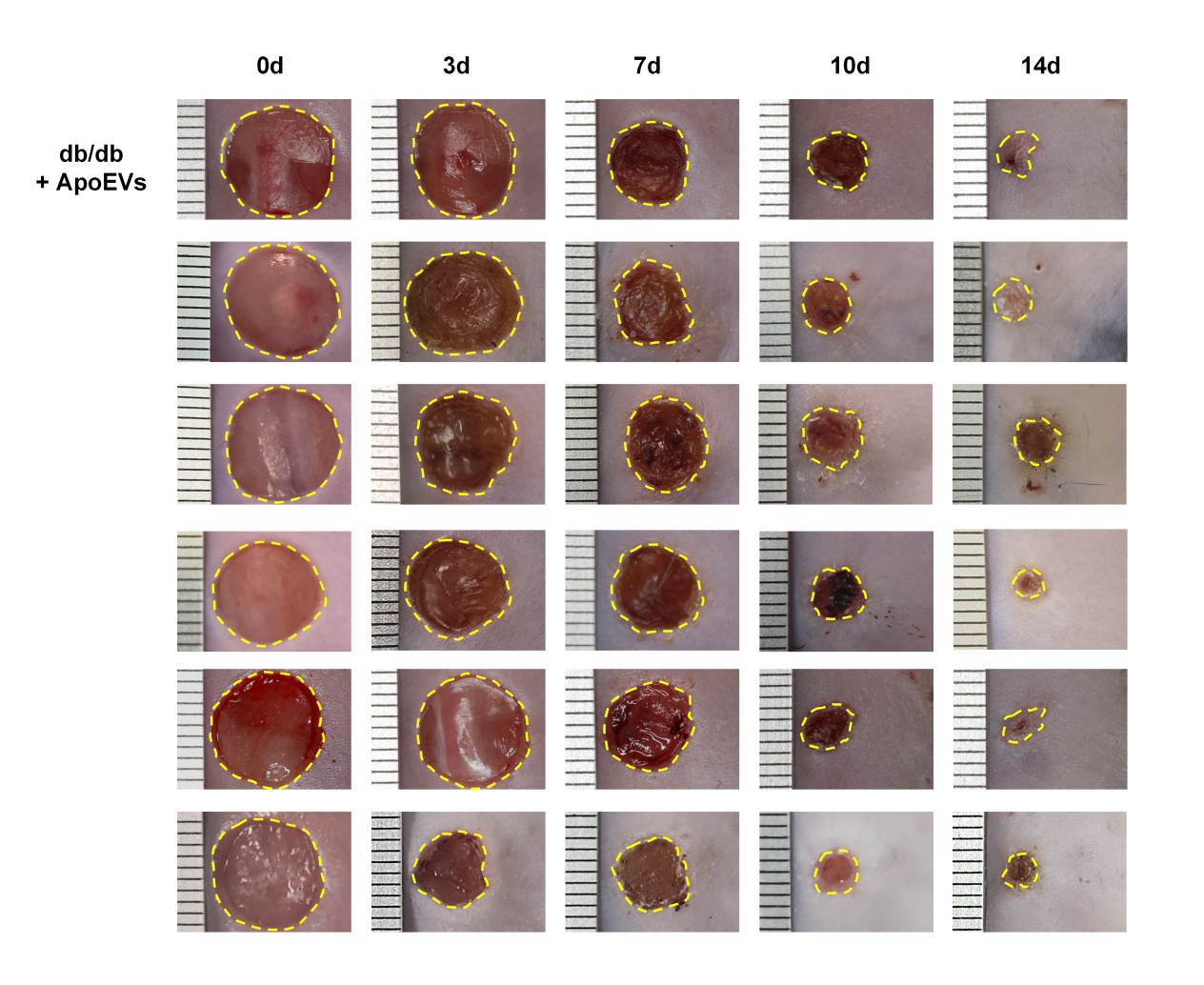
**

Supplement: Supplementary file 3 — Additional file 3: Other photographs of cutaneous wounds not shown in the figure. [file 13287_2023_3490_MOESM3_ESM.docx]
