# Supplementary material for: Umbilical cord mesenchymal stem cell-derived apoptotic extracellular vesicles ameliorate cutaneous wound healing in type 2 diabetic mice via macrophage pyroptosis inhibition
Source: Stem Cell Res Ther. 2023 Sep 19;14:257. doi: 10.1186/s13287-023-03490-6 (PMC10510296; doi:10.1186/s13287-023-03490-6)

**Additional File 4**

**Images of histological staining of other slices not shown in the figure.**

**A Images of the H&E staining of the skin samples.**


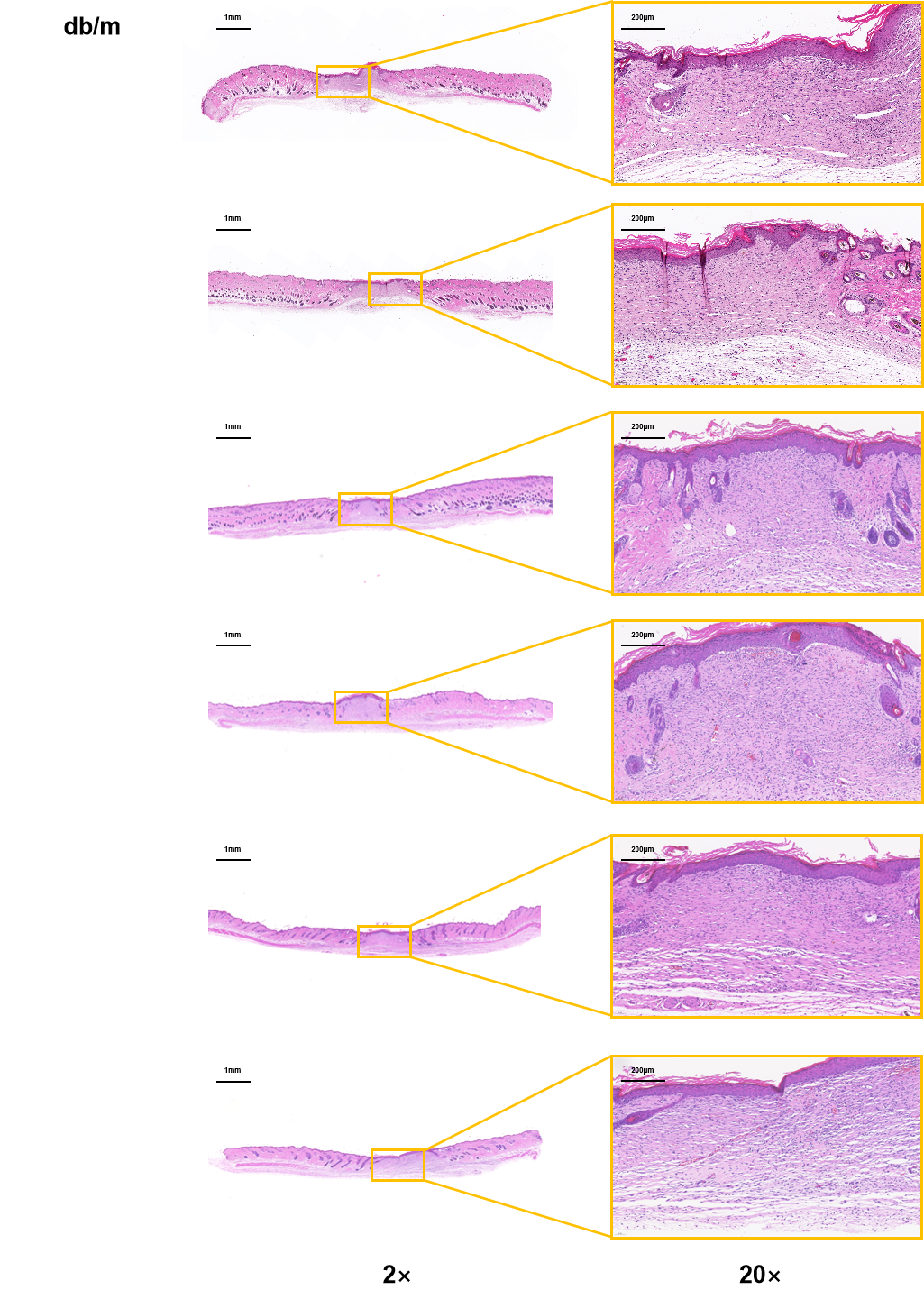


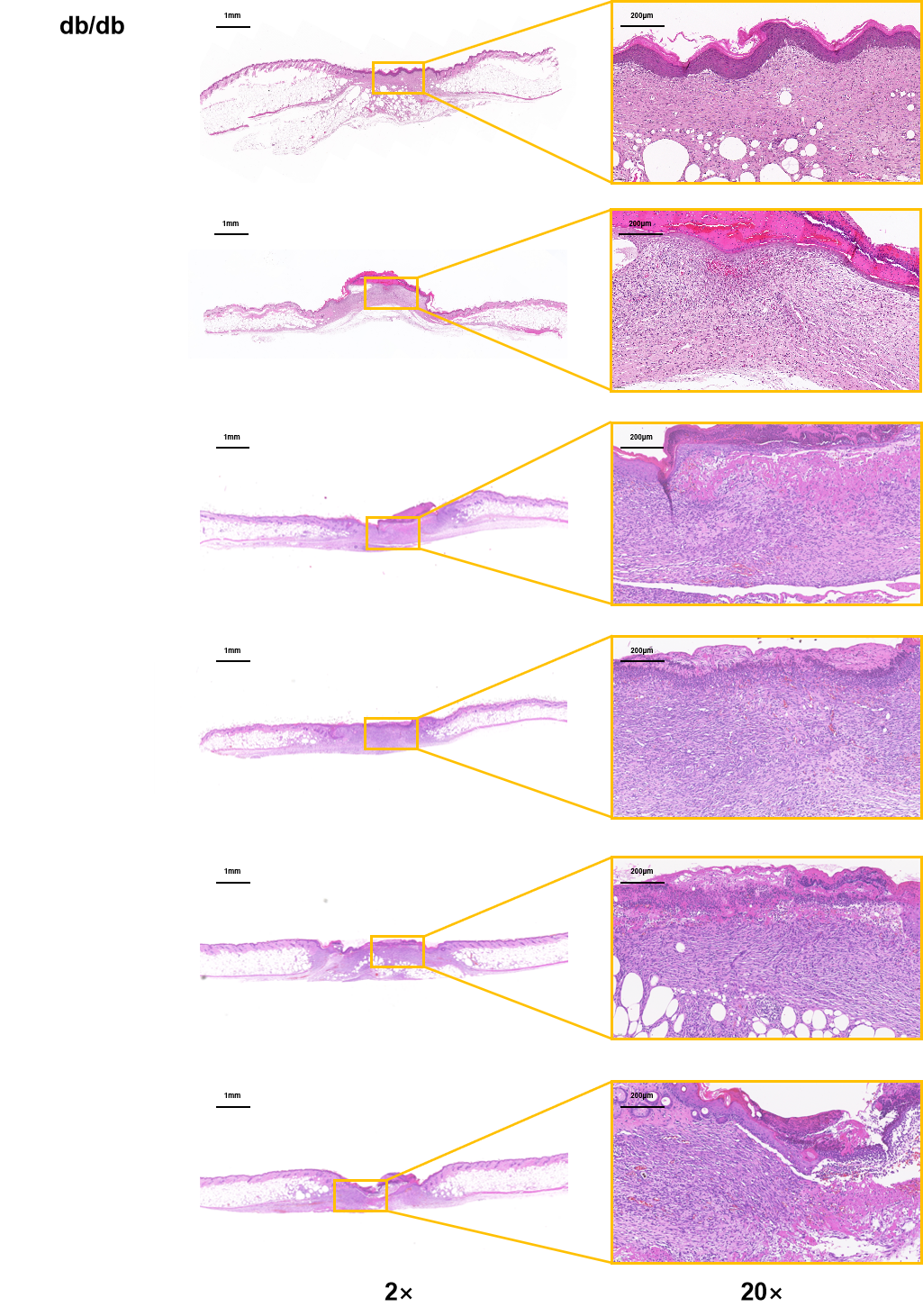


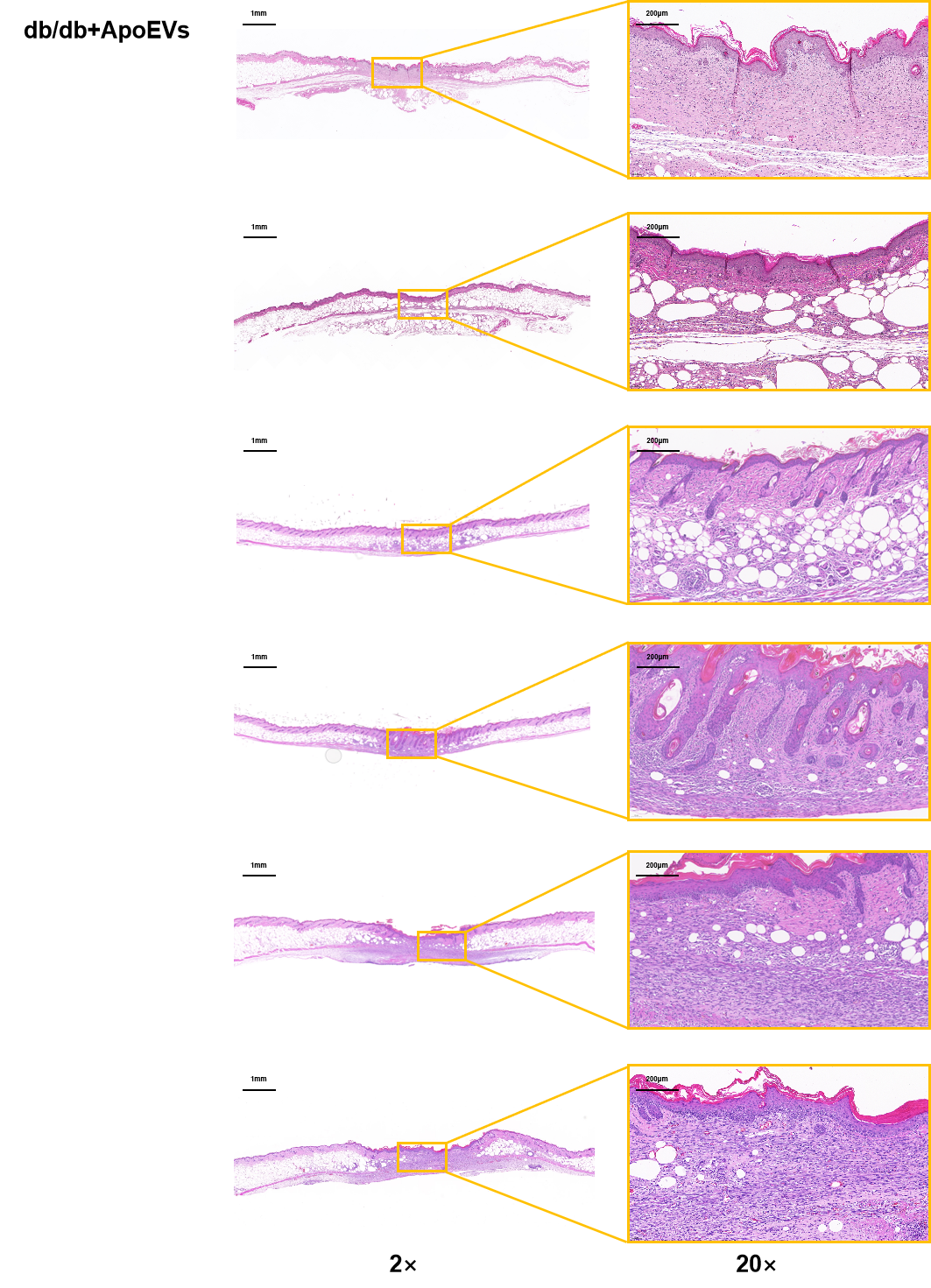


**B Images of the Masson staining of the skin samples.**


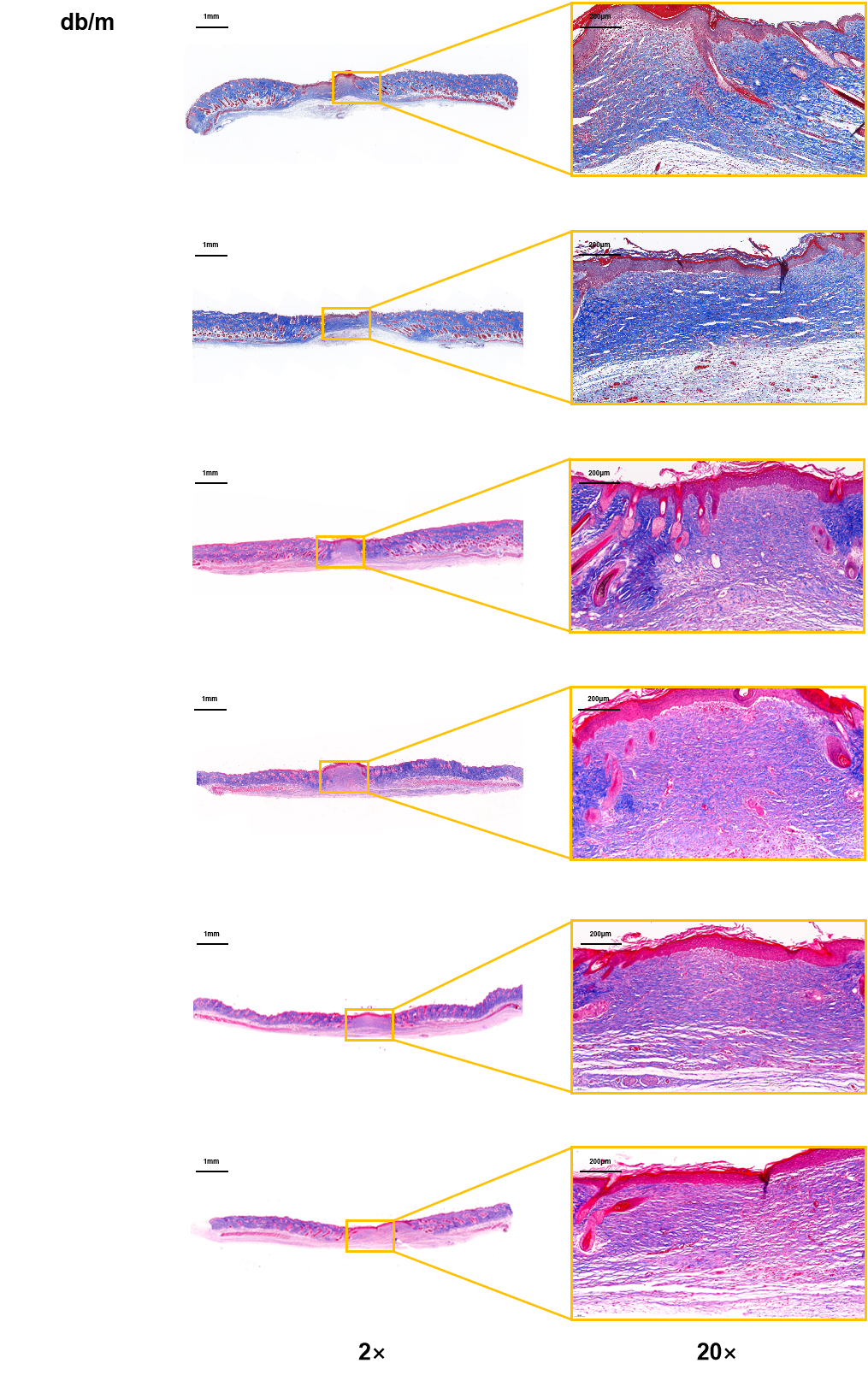


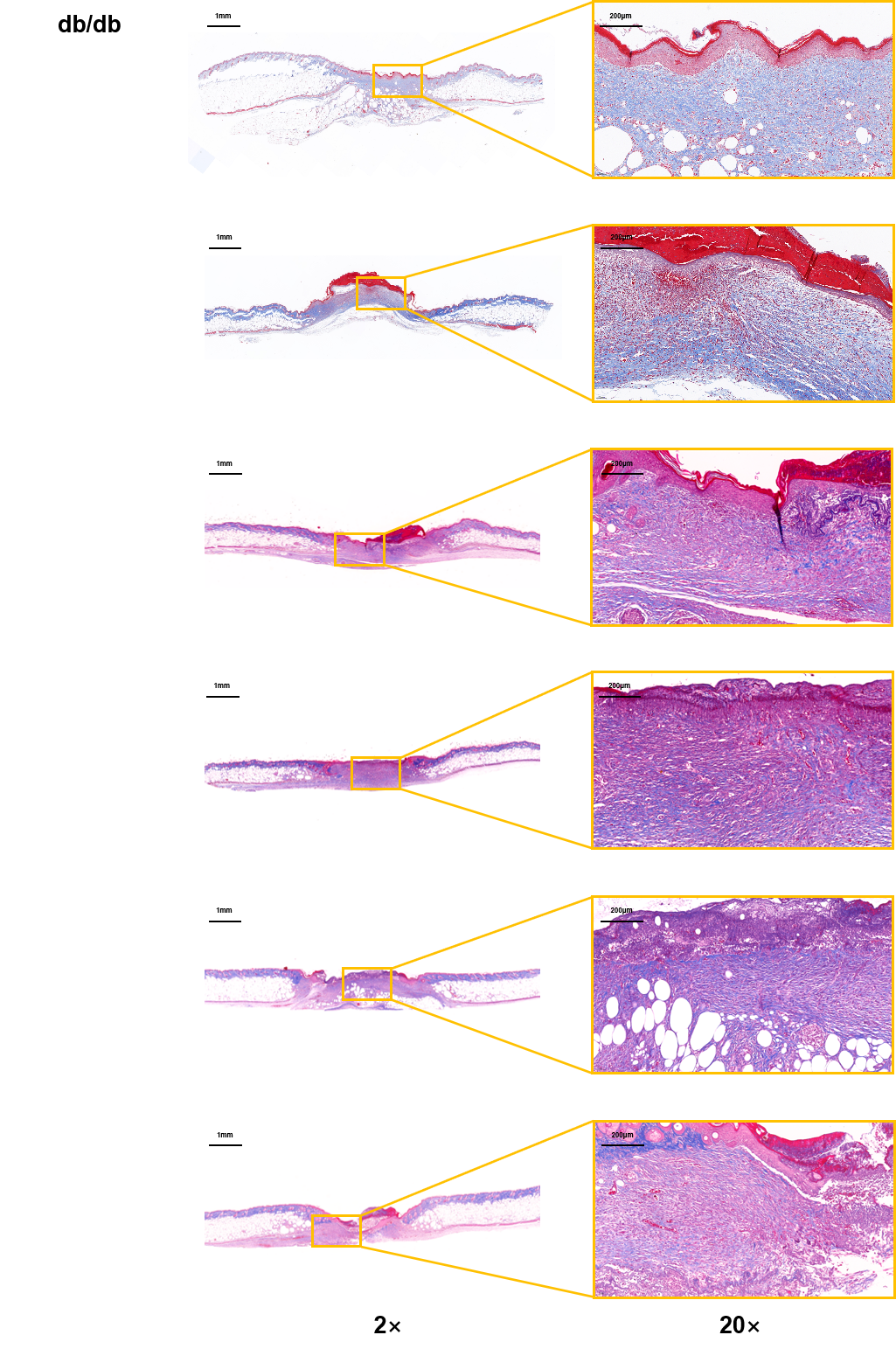


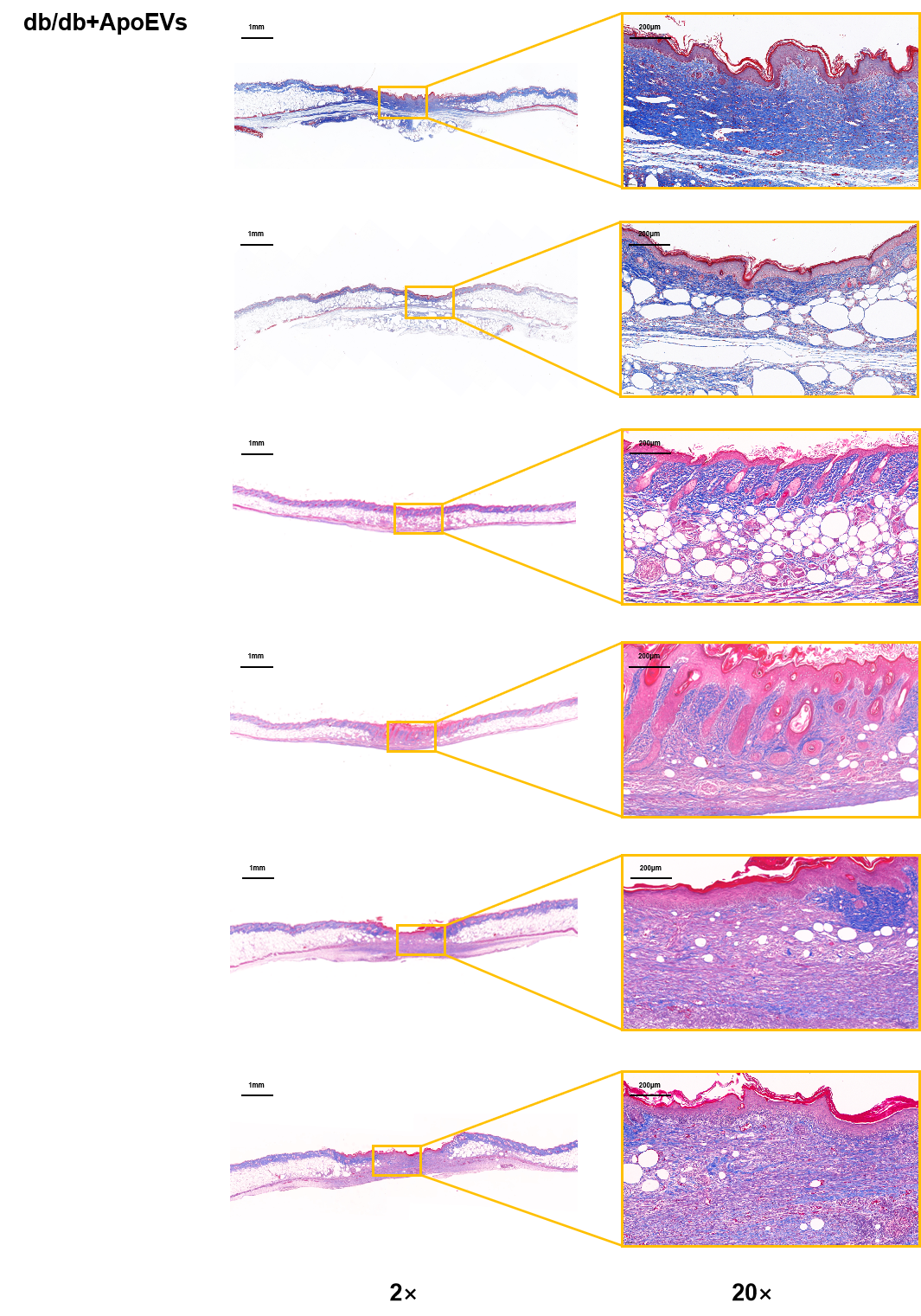

Supplement: Supplementary file 4 — Additional file 4: Images of histological staining of other slices not shown in the figure. a Images of the H&E staining of the skin samples. Scale bar, 1 mm in low-magnification images, 100 μm in high-magnification images. b Images of the Masson staining of the skin samples. Scale bar, 1 mm in low-magnification images, 200 μm in high-magnification images. [file 13287_2023_3490_MOESM4_ESM.docx]
