# Supplementary material for: Umbilical cord mesenchymal stem cell-derived apoptotic extracellular vesicles ameliorate cutaneous wound healing in type 2 diabetic mice via macrophage pyroptosis inhibition
Source: Stem Cell Res Ther. 2023 Sep 19;14:257. doi: 10.1186/s13287-023-03490-6 (PMC10510296; doi:10.1186/s13287-023-03490-6)

**Additional File 5**

**Corresponding uncropped full-length gels and blots.**

**A Corresponding uncropped full-length gels and blots of Fig. 1G.**


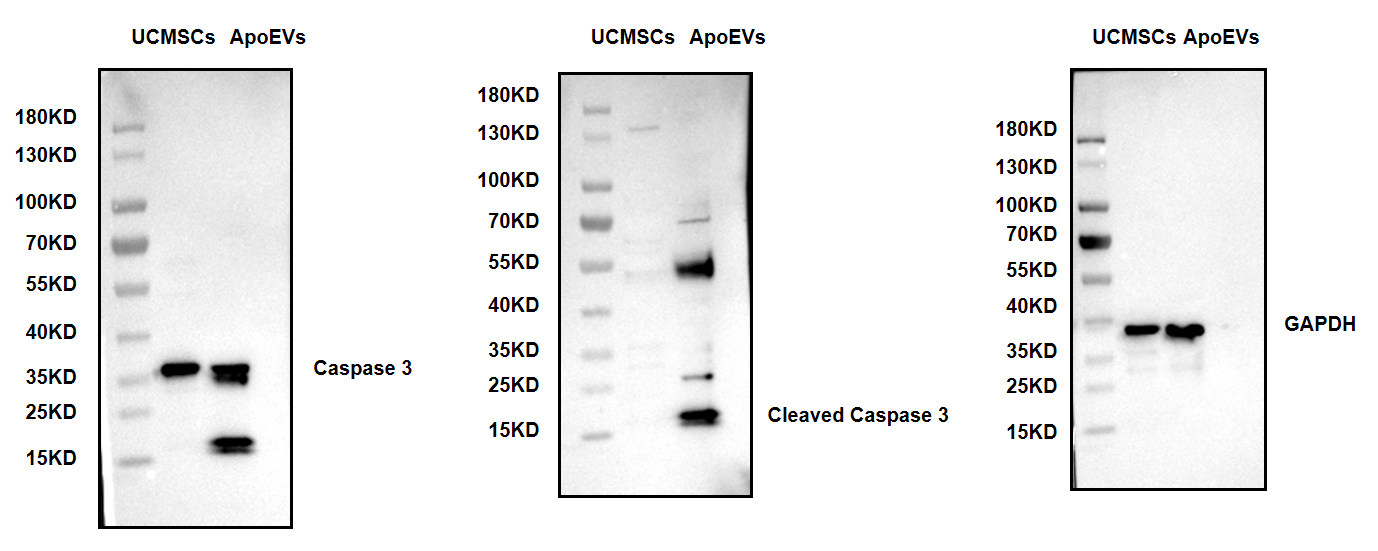


**B Corresponding uncropped full-length gels and blots of Fig. 3C.**


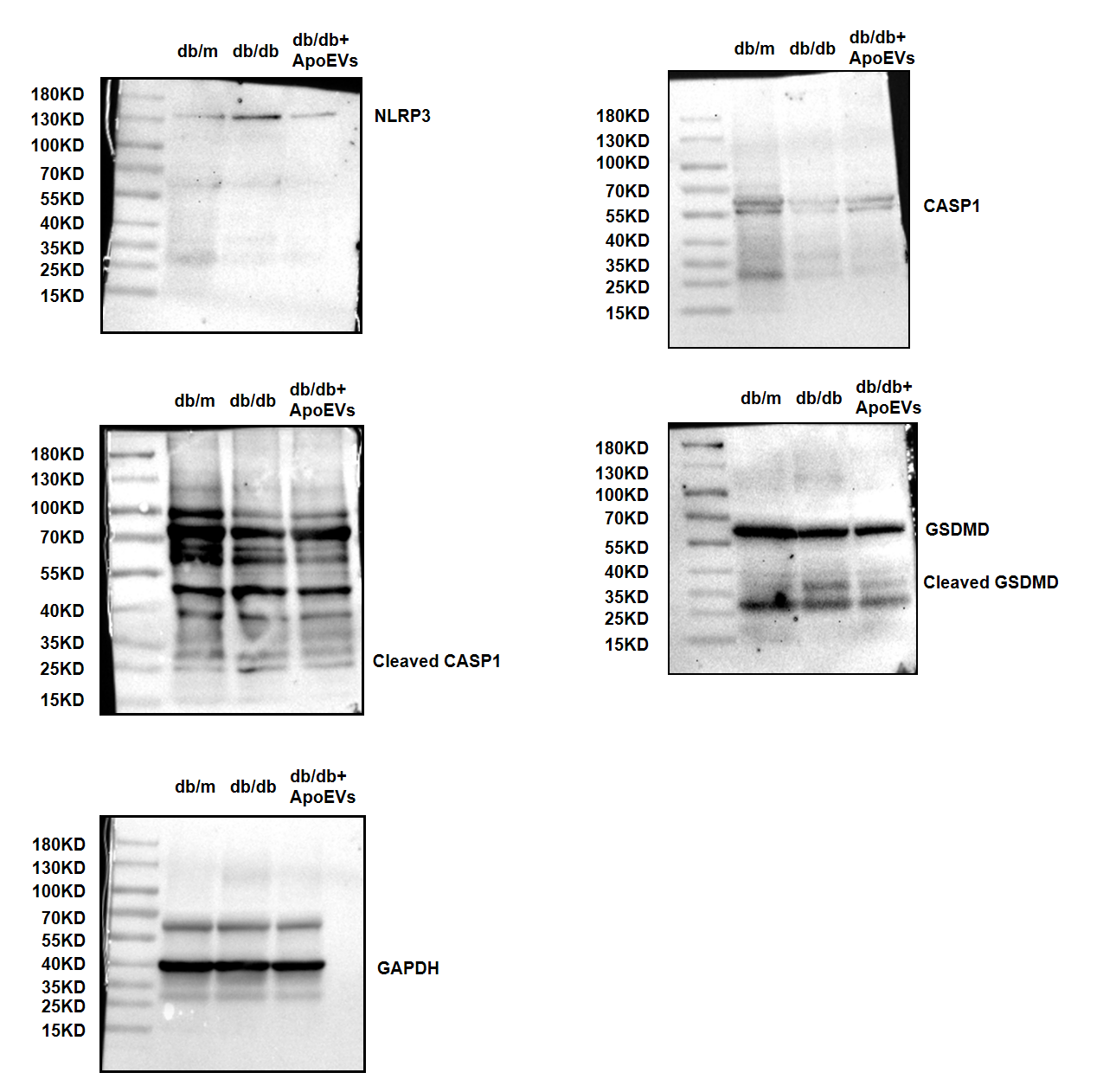


**C Corresponding uncropped full-length gels and blots of Fig. 4E.**


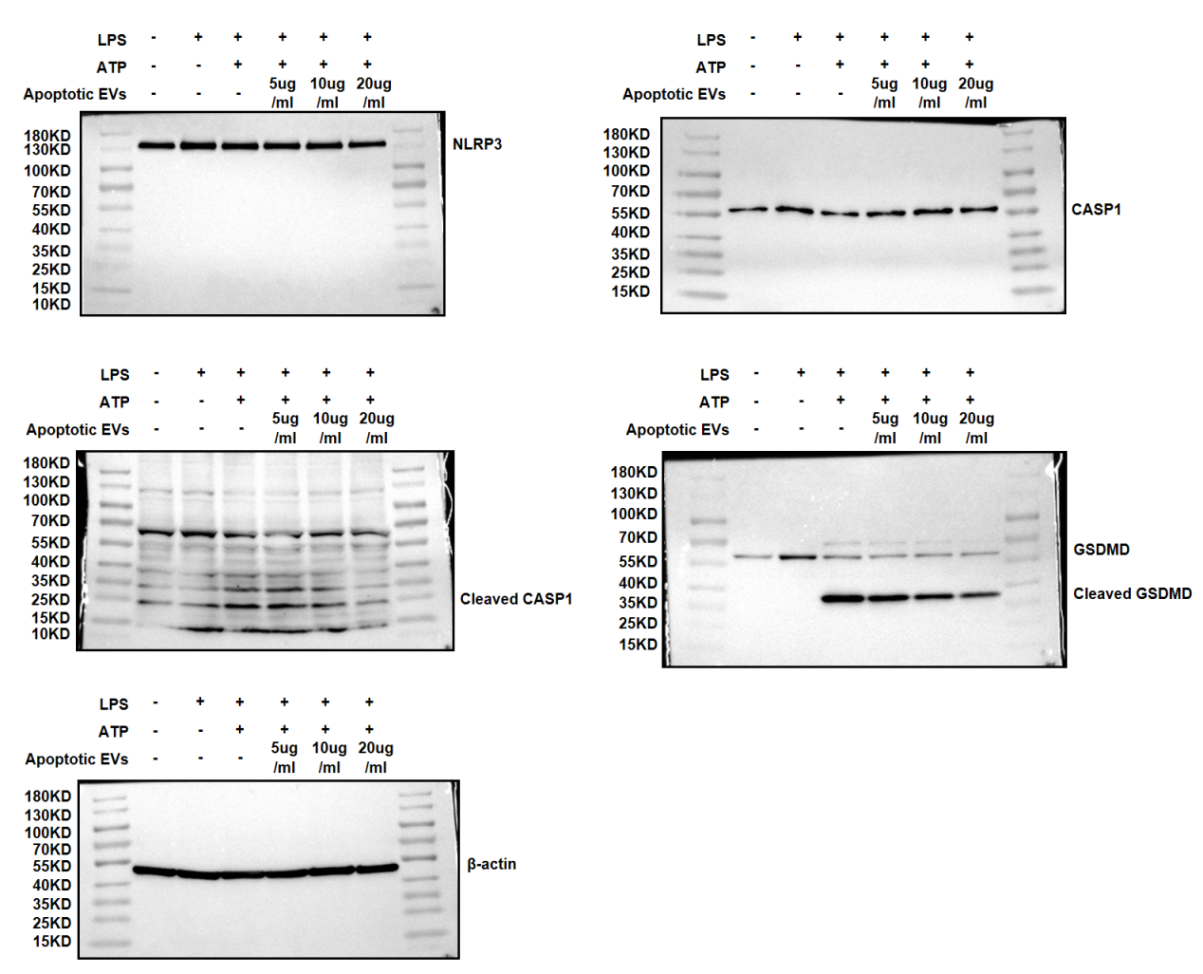


**D Corresponding uncropped full-length gels and blots of Fig. 5C.**


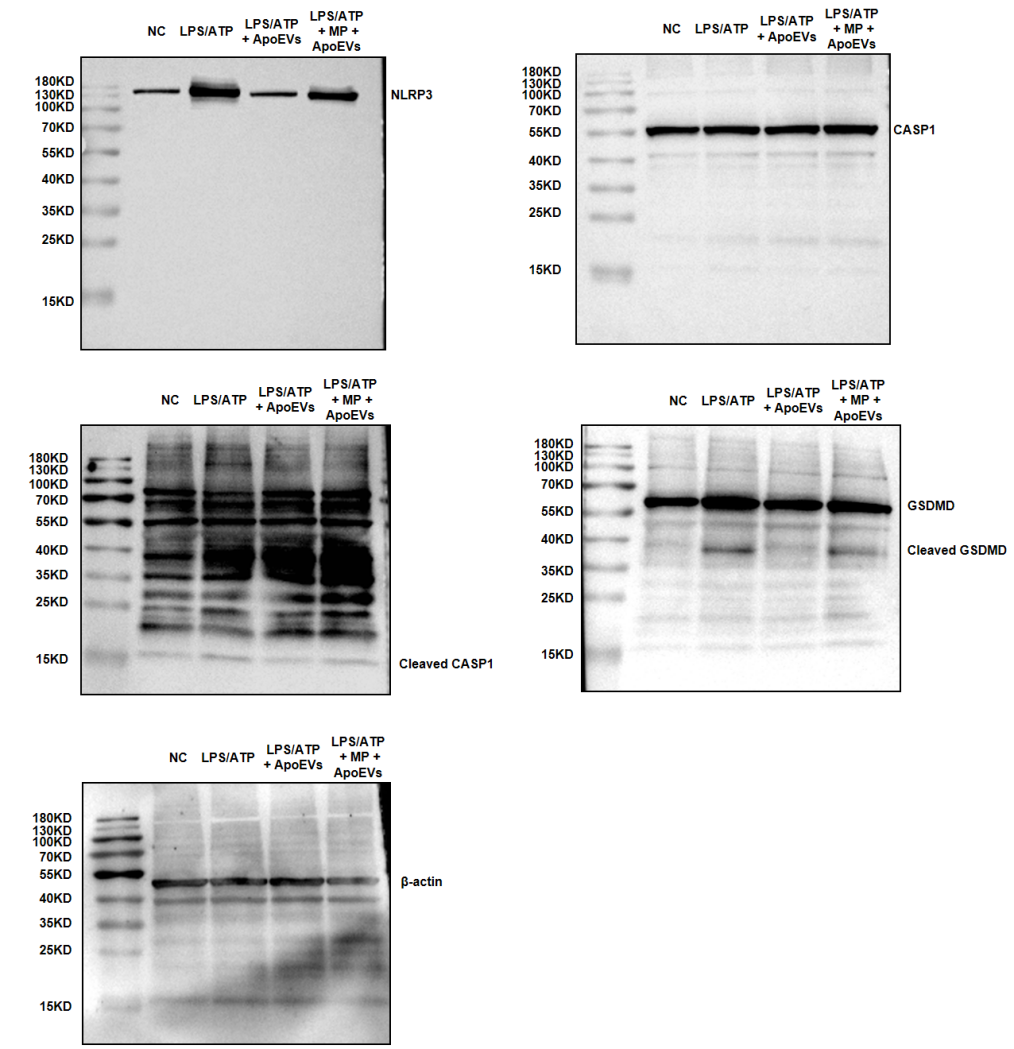


**E Other corresponding uncropped full-length gels and blots of pyroptosis inhibition in vivo not shown in the figure.**


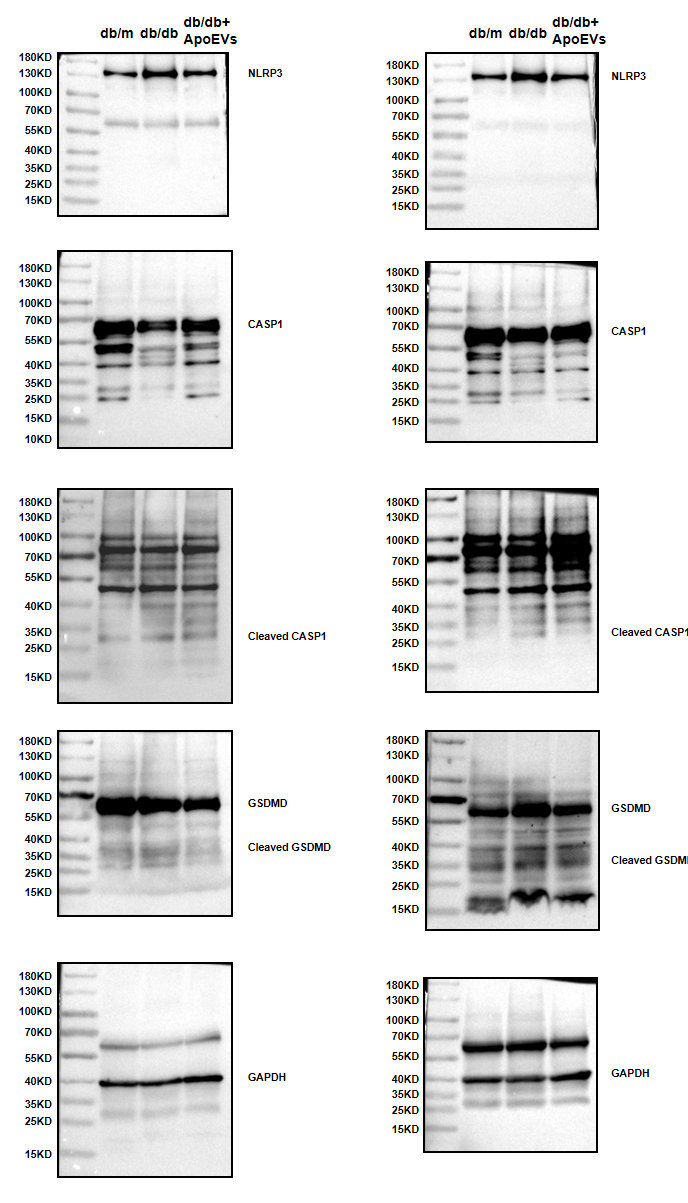


**F Other corresponding uncropped full-length gels and blots of pyroptosis inhibition in vitro not shown in the figure.**


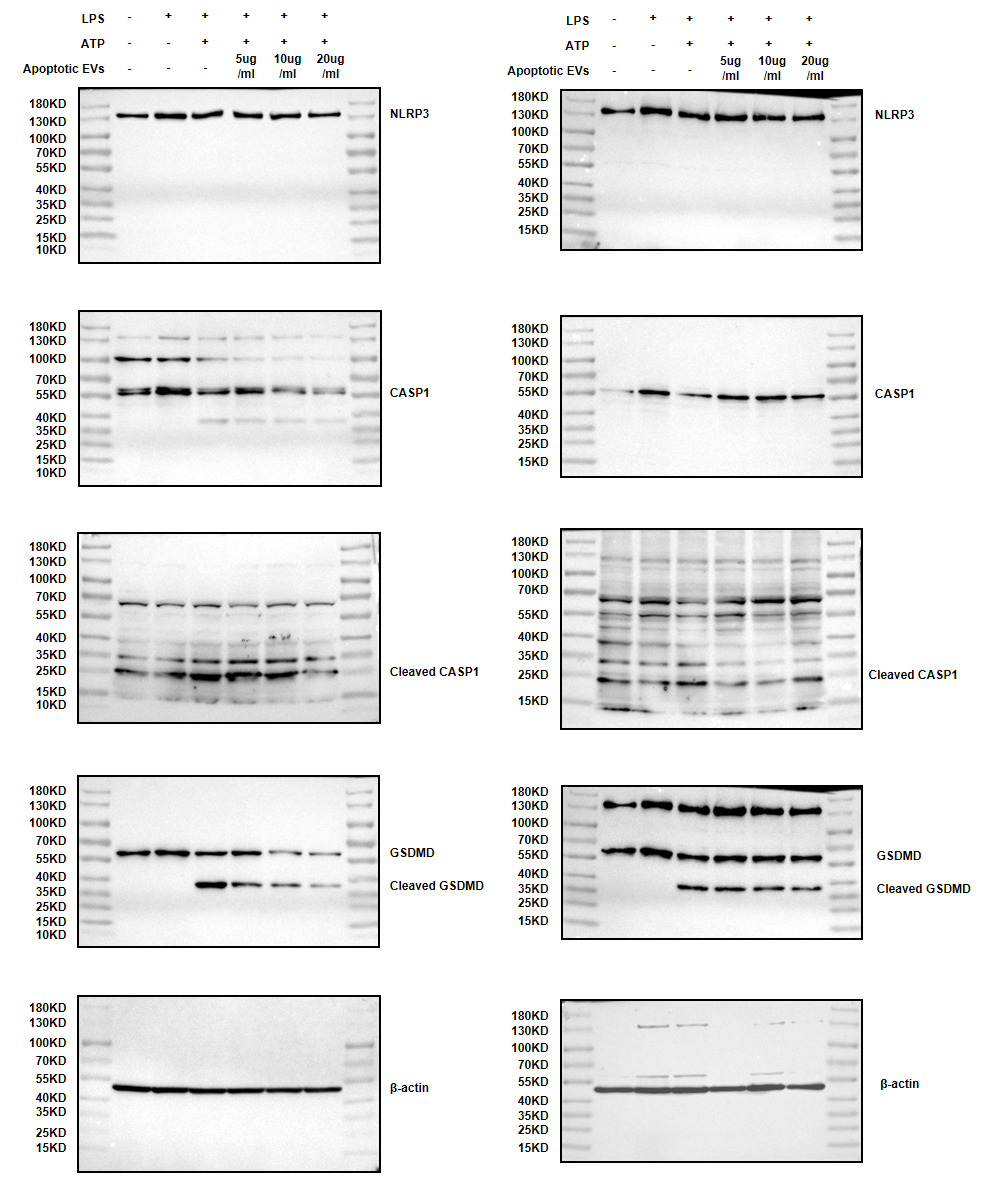


**G Other corresponding uncropped full-length gels and blots of methyl palmitate intervention not shown in the figure.**


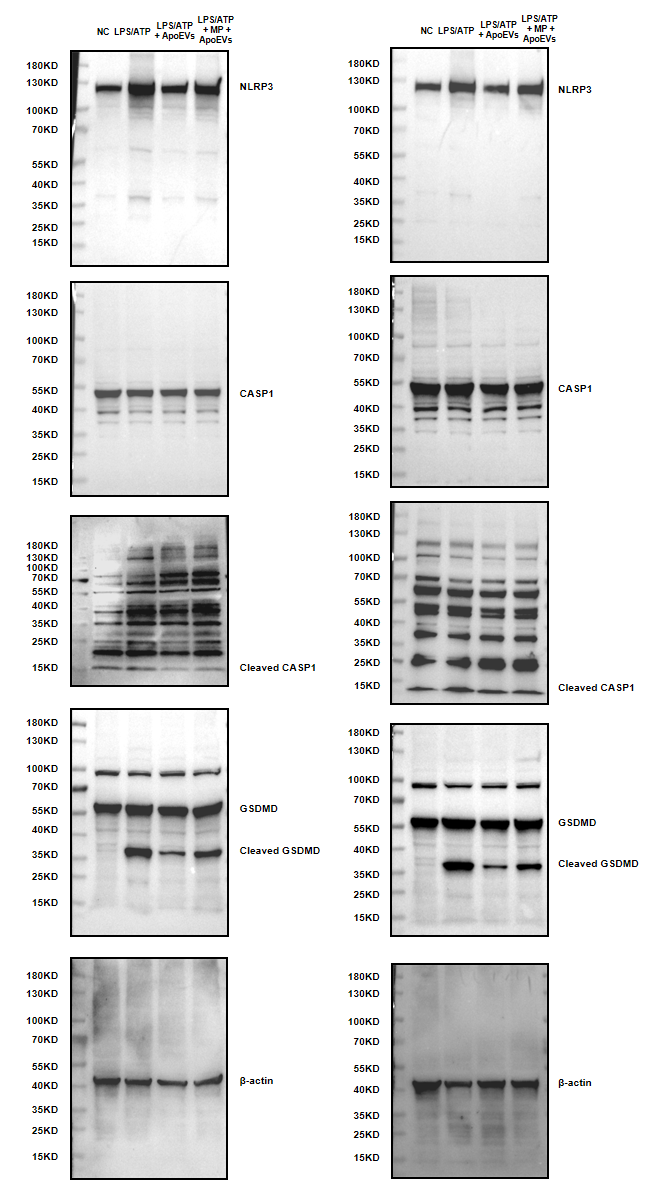

Supplement: Supplementary file 5 — Additional file 5: Corresponding uncropped full-length gels and blots. a Corresponding uncropped full-length gels and blots of Fig. 1G. b Corresponding uncropped full-length gels and blots of Fig. 3C. c Corresponding uncropped full-length gels and blots of Fig. 4E. d Corresponding uncropped full-length gels and blots of Fig. 5C. e Other corresponding uncropped full-length gels and blots of pyroptosis inhibition in vivo not shown in the figure. f Other corresponding uncropped full-length gels and blots of pyroptosis inhibition in vitro not shown in the figure. g Other corresponding uncropped full-length gels and blots of methyl palmitate intervention not shown in the figure. [file 13287_2023_3490_MOESM5_ESM.docx]
